# Systems Analysis of miRNA-Mediated Host Regulatory Response in HPV-Associated Cervical Malignancy

**DOI:** 10.34133/csbj.0031

**Published:** 2026-04-03

**Authors:** Ishrat Khan, NS Suneesh, R Harshithkumar, Ashwini More, Shyam Sundar Nandi, Abdul Arif Khan, Anupam Mukherjee

**Affiliations:** ^1^ ICMR–National Institute of Virology, Pune 400001, Maharashtra, India.; ^2^ Department of Biotechnology, Savitribai Phule Pune University, Pune 411007, MH, India.; ^3^ AcSIR–Academy of Scientific & Innovative Research, Ghaziabad 201002, UP, India.; ^4^ ICMR–National Institute of Translational Virology and AIDS Research, Pune 400026, Maharashtra, India.

## Abstract

Persistent high-risk human papillomavirus (HPV) infection, particularly types 16 and 18, is the primary driver of cervical carcinogenesis. These viruses utilize viral oncoproteins to manipulate host gene expression through multiple regulatory mechanisms. Within this landscape, microRNAs (miRNAs) emerge as critical posttranscriptional modulators that contribute to the dysregulation of pathways involved in cellular transformation and tumor progression. In this study, we investigated HPV-associated miRNA dysregulation using HPV-negative (C33A) and HPV-positive (SiHa, HeLa) cervical cancer cell lines, integrating custom microarray profiling with comprehensive systems biology and bioinformatic analyses. Our results identified 42 dysregulated miRNAs, including hsa-miR-125b-5p, hsa-miR-106b-5p, hsa-miR-23b-3p, and hsa-miR-30d-5p, which were significantly down-regulated across all experimental models. Integration of these miRNAs with cervical carcinoma transcriptomic data (GSE151666) revealed that HPV16 and HPV18 distinctly remodel host gene networks to drive malignancy. Network analysis pinpointed specific regulatory hubs, such as *SOX2* and *SERPINE1* for HPV16, and *ERBB4* and *GLI1* for HPV18, which may facilitate malignant tumor growth by disrupting metabolic, keratinization, and extracellular matrix pathways. Furthermore, drug–gene interaction mapping highlighted potential therapeutic targets including *CDKN2A*, *CD274*, and *GLI1*. These findings suggest that different HPV subtypes may employ unique miRNA-mediated strategies to promote cancer, offering new avenues for the development of precise diagnostic biomarkers and targeted therapies within cervical cancer research.

## Graphical Abstract

**Figure:**
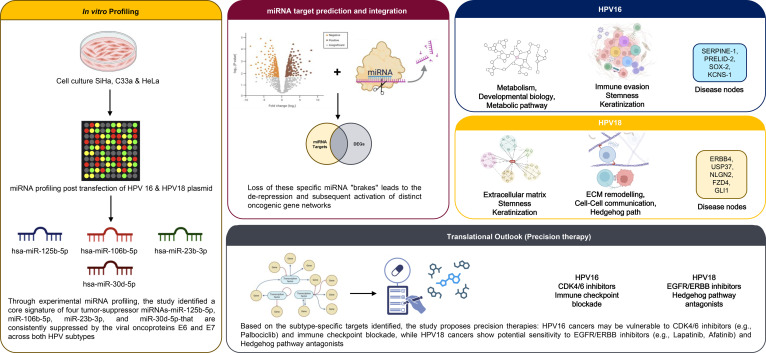

## Introduction

Human papillomavirus (HPV) is a small, non-enveloped, double-stranded DNA virus of approximately 8,000 base pairs belonging to the *Papillomaviridae* family that infects epithelial tissues, causing transient infections, benign lesions, anogenital warts, and, in some cases, progression to cancer [1]. More than 200 HPV subtypes have been identified and classified as low risk (LR) or high risk (HR) based on oncogenic potential [2]. While LR-HPVs such as types 6 and 11 are associated with benign lesions, HR-HPVs, including types 16, 18, 31, 33, and 45, are responsible for most anogenital and oropharyngeal cancers, with HPV16 and HPV18 accounting for nearly 70% of cervical cancers worldwide [3]. Although most infections are cleared within 12 months, persistent HR-HPV infection promotes malignant transformation, particularly when viral DNA integrates into the host genome, leading to deregulated expression of E6 and E7 oncogenes [4].

E6 and E7 disrupt multiple cellular processes, including cell cycle control, DNA repair, apoptosis, chromosomal stability, and immune recognition. E6 targets p53 for proteasomal degradation [5], whereas E7 inactivates pRb, releasing E2F transcription factors (TFs) and driving uncontrolled proliferation [6]. These events, along with immune evasion and epigenetic dysregulation, create a pro-tumorigenic environment in infected epithelial cells [7]. HPV infection rarely induces early inflammation due to minimal cytolysis and the absence of viremia; however, persistent infection gradually alters cytokine secretion and immune infiltration [8]. Progression to malignancy is influenced by viral, host, and environmental factors, including epigenetic reprogramming of tumor suppressors and oncogenes [9].

While the direct interactions of E6 and E7 with host proteins like p53 and pRb are central to oncogenesis, the resulting cellular environment is further shaped by indirect shifts in host microRNA (miRNA) networks. These miRNAs function as significant components of the larger regulatory circuitry, fine-tuning the expression of genes involved in immune evasion and cell cycle control downstream of primary viral induced signaling changes. miRNAs, small noncoding RNAs of 19 to 25 nucleotides, regulate gene expression posttranscriptionally through incomplete pairing with 3′ untranslated regions of target mRNAs. They modulate diverse processes, including cell cycle regulation, autophagy, apoptosis, inflammation, metastasis, and chemoresistance [10–13]. Dysregulation of miRNAs has been reported in HPV-associated cervical carcinogenesis [14–16], with several miRNAs such as miR-34a, miR-9, miR-21, and miR-145 linked to cellular transformation [17,18]. Given their regulatory potential, miRNAs represent promising biomarkers and therapeutic targets in HPV-associated cancer. The differential expression of miRNAs across various HPV-positive cell lines was notably examined in uterine cervical cancer, demonstrating that HPV status and subtype significantly influence global miRNA profiles [19]. However, the downstream functional architecture and therapeutic targetability of these subtype-specific networks remain to be fully elucidated.

Beyond the well-documented “direct” interference of E6 and E7 with core host proteins such as p53 and pRb, there is a more nuanced, systemic layer of viral influence. Our study focuses on the miRNA-mediated axis, which we view as a parallel regulatory mechanism that the virus subverts to achieve long-term host remodeling. By characterizing this layer, we provide insight into how HPV not only disrupts cellular checkpoints but also fundamentally reprograms the host’s regulatory landscape to sustain malignancy. In this study, we profiled cellular miRNA expression in HPV-negative (C33A) and HPV-positive (SiHa and HeLa) cervical cancer cells, as well as in HPV-transfected C33A models. We further integrated in vitro expression profiles with Gene Expression Omnibus (GEO) and The Cancer Genome Atlas (TCGA) datasets to identify miRNA-regulated gene networks, hub targets, pathway perturbations, and drug–gene interactions. The objective of this study is to provide a comprehensive map of the regulatory landscape governing host miRNA-mediated HPV-associated malignancy. With recent advances in RNA-based therapeutics, delineating miRNA-mediated regulation in HPV infection may accelerate the development of targeted therapy and biomarker strategies aimed at persistent infection and cervical malignancy.

## Materials and Methods

### Cell culture

Human cervical cancer cell lines C33A (HPV-negative), SiHa (HPV16-positive), and HeLa (HPV18-positive) were obtained from the National Centre for Cell Science (Pune, India). The C33A cell line was utilized as an HPV-negative carcinoma reference. While C33A possesses intrinsic genetic alterations common to cervical malignancy (such as p53 mutations), its use as a comparator allows for the distinction between general oncogenic dysregulation and the specific miRNA-mediated signatures driven by HPV16 and HPV18 oncoproteins. Cultures were routinely examined for morphology, mycoplasma contamination, and authentication. Cells were maintained in Dulbecco’s modified Eagle’s medium (DMEM) (Gibco, USA) supplemented with 10% fetal bovine serum (FBS) (Gibco, USA), 1% penicillin–streptomycin (Sigma-Aldrich, USA), and 20 mM Hepes (Gibco, USA). Cultures were incubated at 37 °C with 5% CO₂ and subcultured at approximately 80% confluency for downstream applications.

### miRNA microarray profiling

To examine miRNA dysregulation associated with HPV infection, total miRNA was profiled using miRCURY LNA Cancer-Focus custom polymerase chain reaction (PCR) arrays (YAHS-102Y:339325, Qiagen, Germany). Total miRNA was extracted using the mirVana miRNA Isolation Kit (Invitrogen, USA; catalog no. AM1560), and 10 ng of RNA was reverse transcribed using the miRCURY LNA RT Kit (Qiagen, Germany). Quantitative PCR (qPCR) was performed using the miRCURY LNA SYBR Green Master Mix (Qiagen, Germany) under the following conditions: 95 °C for 2 min, followed by 40 cycles of 95 °C for 10 s and 56 °C for 60 s. Fold-change values were calculated using the GeneGlobe analysis tool (https://geneglobe.qiagen.com; accessed on 2025 March 31). Differential expression was defined as fold change ≥ 2.0 or ≤ −2.0 with *P* < 0.05 from 3 independent biological replicates.

### Plasmids and transient transfection

Plasmids carrying HPV oncogenes included p1321 HPV-16 E6/E7 (plasmid #8641), MSCV-C 18E7 (plasmid #85035), and full-length HPV16 [American Type Culture Collection (ATCC) 45113] and HPV18 (ATCC 45152) genomes. C33A cells were cultured to ~50% confluence and incubated in serum-free medium for 1 h prior to transfection. Cells were transfected using Lipofectamine 2000 (Invitrogen; catalog no. 11668019) according to the manufacturer’s instructions. After 6 h, serum-free medium was replaced with DMEM containing 10% FBS, and cells were harvested after 48 h. Transfection efficiency was verified by SYBR-based qPCR targeting HPV16 E7 and HPV18 E7, with glyceraldehyde-3-phosphate dehydrogenase (GAPDH) as an internal control. All transfection experiments were performed in 3 independent biological replicates.

### Validation of miRNA expression by TaqMan qRT-PCR

Four miRNAs showing significant dysregulation in microarray analysis were selected for validation: hsa-miR-125b-5p (assay ID: 000449), hsa-miR-106b-5p (assay ID: 000442), hsa-miR-23b-3p (assay ID: 000400), and hsa-miR-30d-5p (assay ID: 000420). Total RNA was isolated using TRIzol reagent (Invitrogen, USA; catalog no. 15596026). cDNA synthesis and qPCR were performed using Applied Biosystems TaqMan MicroRNA Assays (4427975). RNA-U6 served as an endogenous control, and relative expression was calculated using the 2^−ΔΔCt^ method. Quantitative reverse transcription PCR (qRT-PCR) experiments were performed with a minimum of 3 independent biological replicates, each analyzed with 3 technical replicates. Results are expressed as mean ± SD. Statistical analyses were performed using GraphPad Prism (version 5, GraphPad Software, USA). Differences between groups were evaluated using one-way analysis of variance (ANOVA) followed by Tukey’s multiple comparison test, and a *P* value of <0.05 was considered statistically significant.

### miRNA target prediction

Target genes for the selected dysregulated miRNAs were identified using miRWalk 3.0 (http://mirwalk.umm.uni-heidelberg.de/) [20]. The top 4 dysregulated miRNAs selected from the microarray analysis were used as query inputs. The corresponding target genes of these miRNAs were retrieved from the database and downloaded for subsequent integration and functional analyses.

### Dataset selection and differential expression analysis

To correlate miRNA dysregulation with HPV-associated gene expression changes, RNA-Seq dataset GSE151666 from the GEO database (https://www.ncbi.nlm.nih.gov/geo/) was analyzed. This dataset includes transcriptomic profiles from HPV16- and HPV18-positive cervical cancer samples. For this analysis, samples were stratified exclusively by HPV genotype (HPV16 versus HPV18) to capture virus-specific regulatory patterns; further stratification was not performed to maintain adequate statistical power for the differential expression analysis. Differential expression analysis was performed using GEO2R (https://www.ncbi.nlm.nih.gov/geo/geo2r/), which uses DESeq2 [21]. Significantly altered genes were defined using false discovery rate (FDR) < 0.05 and an absolute log₂ fold change > 1.5. Only up-regulated genes were selected for integration with miRNA target predictions, based on the assumption that down-regulated miRNAs release repression on their targets. Overlapping genes between predicted targets (miRWalk) and differentially expressed genes (DEGs) were identified through Venn analysis for HPV16 and HPV18 separately.

### Gene Ontology for molecular function analysis

Gene Ontology (GO) molecular function (MF) enrichment was performed on overlapping target genes using DAVID v2025_1 [22,23]. Gene lists were analyzed separately for HPV16 and HPV18, using *Homo sapiens* as background and Benjamini–Hochberg correction (adjusted *P* < 0.05). In addition to GO categories, UniProt keywords were included to capture broader functional similarities. Enriched terms were visualized using semantic clustering and bubble plots generated through ggplot2, highlighting functional differences associated with HPV subtype-specific miRNA deregulation.

### Pathway enrichment analysis

Biological pathway enrichment was conducted using DAVID across Kyoto Encyclopedia of Genes and Genomes (KEGG), Reactome, and WikiPathways databases. Separate analyses were performed for HPV16 and HPV18 to identify subtype-specific signaling perturbations (significance threshold: *P* < 0.05). The intersecting and unique pathways across both subtypes were visualized using UpSetR and ComplexUpset plots. Emphasis was placed on pathways related to metabolic regulation, keratinization, extracellular matrix (ECM) remodeling, and cancer-associated signaling.

### TF enrichment and protein–protein interaction network

To identify transcriptional regulators associated with miRNA target genes, TF enrichment was performed using DAVID with a Benjamini–Hochberg FDR < 0.05. Protein–protein interaction (PPI) networks for HPV16- and HPV18-associated genes were then constructed using STRING v12.0 with a confidence threshold ≥ 0.7. Networks were visualized in Cytoscape to map functional clusters and regulatory modules influenced by miRNA dysregulation in HPV infection. While initial network construction was seeded with up-regulated GEO-derived genes overlapping with miRNA targets, subsequent PPI expansion and topological analyses enabled the inclusion of highly connected regulatory nodes regardless of their expression levels.

### Network topology and hub gene identification

Network topology analysis was performed using the CytoHubba plugin in Cytoscape to prioritize central regulatory genes in HPV16 and HPV18 networks. Multiple parameters, including degree, betweenness, clustering coefficient (CC), and shortest path length, were applied to reduce ranking bias. Disease node scores were calculated in R to generate weighted hub gene lists. Top candidates were represented in lollipop plots, which identified potential drivers of cervical carcinogenesis. The regulatory significance of network nodes was further evaluated by computing a composite disease node score based on multiple topological parameters, including degree, betweenness centrality (BC), CC, and average shortest path length (ASPL). These 4 metrics were selected and weighted equally to capture distinct, complementary biological dimensions of network influence: Degree identifies local regulatory hubs, BC highlights informational bottlenecks, CC indicates modular density, and ASPL measures global network reachability. Furthermore, because the constructed HPV16 and HPV18 networks differ substantially in size and density, relying on raw topological values would inherently bias hub rankings toward the larger network. To ensure comparability across metrics and mitigate this sensitivity to network size differences, individual network parameters were normalized using min–max normalization. For parameters positively associated with node importance (degree, BC, and CC), normalization was performed using the following formula:

$$\text{Norm}\left(X_{i}\right)=\frac{X_{i}-X_{\min}}{X_{\max}-X_{\min}}$$ (1)

where *X_i_* represents the parameter value for node *i*, and *X_min_* and *X_max_* denote the minimum and maximum values of that parameter across the network. For ASPL, which inversely correlates with node importance, normalization was performed as follows:

$$\text{Norm}\left(\mathrm{ASP}L_{i}\right)=\frac{\mathrm{ASP}L_{\max}-\mathrm{ASP}L_{i}}{\mathrm{ASP}L_{\max}-\mathrm{ASP}L_{\min}}$$ (2)

The disease node score for each gene was then calculated as the average of the normalized parameters. Nodes were ranked by their disease score, and the top-ranking genes were designated as hub nodes for downstream analyses.

### TCGA expression analysis of hub genes

To validate the clinical relevance of the prioritized hub genes, expression profiling was conducted using TCGA-CESC datasets via the UALCAN analysis portal (https://ualcan.path.uab.edu/) [24,25]. The analysis compared mRNA expression levels of each hub gene between normal cervical tissues (*n* = 3) and primary tumor samples (*n* = 305). Expression values were represented as log₂ TPM (transcripts per million). Statistical significance was assessed using Student’s *t* test, and box-whisker plots were generated to display distribution patterns including median, interquartile range, whisker extent, and outlier samples. Expression trends were evaluated for consistency with the predicted directionality of miRNA–mRNA regulation. Genes showing significant expression differences were considered clinically relevant and were prioritized for subsequent pharmacological analysis. Nonsignificant expression patterns were retained for biological interpretation, particularly those associated with tumor microenvironment heterogeneity or subtype-specific effects.

### Network pharmacological analysis for identification of candidate drugs

To explore therapeutic opportunities targeting identified disease hubs, drug–gene interaction profiling was performed using the Drug-Gene Interaction Database (https://dgidb.org/ DGIdb 5.0) [26]. Input genes from both HPV16- and HPV18-associated hubs were queried against curated datasets containing U.S. Food and Drug Administration (FDA)-approved drugs, experimental compounds, and pharmacological annotations from sources including DrugBank (https://go.drugbank.com/), PharmGKB (http://www.pharmgkb.org/), ChEMBL (https://www.ebi.ac.uk/chembl/), TTD (https://idrblab.org/ttd/), and Drug Target Commons (https://drugtargetcommons.fimm.fi/) [27–30]. Only interactions associated with demonstrated anti-neoplastic relevance were prioritized. Additionally, to estimate drug suitability based on genomic alterations, PANDrugs 2 (https://www.pandrugs.org/) analysis was conducted using the same gene set [31,32]. PANDrugs scoring incorporated molecular evidence, mutation status, and reported therapeutic efficacy to generate drug and gene scores. Compounds with high drug score against high-scoring hub genes were shortlisted as candidate therapeutic strategies. Final results highlighted FDA-approved drugs, experimental inhibitors, and natural compounds potentially targeting HPV-associated molecular networks, enabling translational prospects for precision therapy in cervical cancer.

## Results

### HPV infection induces differential expression of miRNAs associated with cancer regulatory pathways in HPV16- and HPV18-positive cervical cell lines

**Fig. 1.**
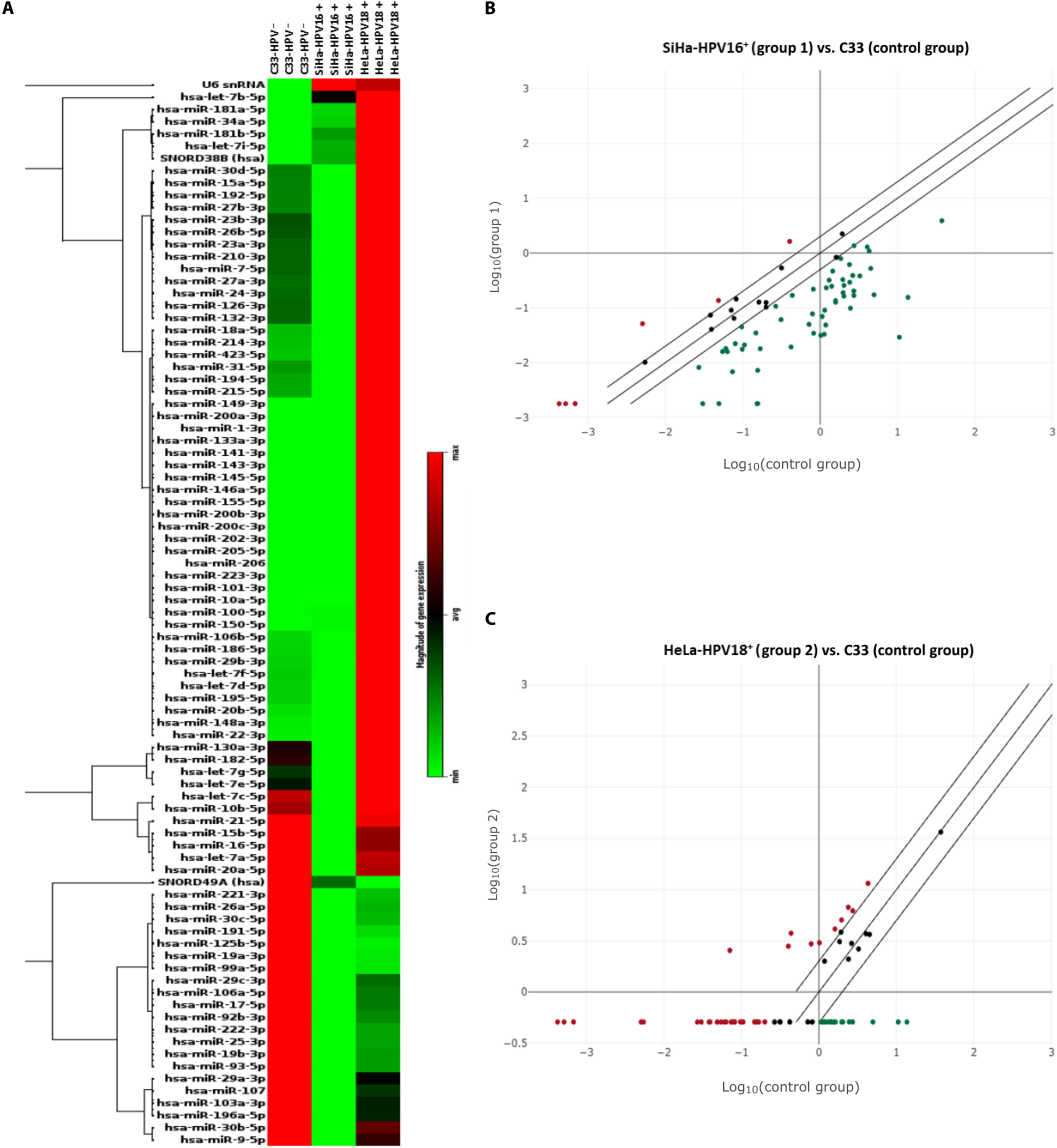
Differential expression of miRNAs in HPV16- and HPV18-positive cervical cancer cell lines. (A) Heat map displaying expression profiles of 84 cancer-associated miRNAs in HPV16-positive SiHa and HPV18-positive HeLa cells compared with HPV-negative C33A cells (control) using the Human Cancer Focus PCR panel. Red indicates maximum expression (up-regulation), while green represents minimum expression (down-regulation). Scatterplots representing differential miRNA expression between HPV-negative C33A and (B) HPV16-positive SiHa or (C) HPV18-positive HeLa cells. Each point denotes a miRNA: Red indicates up-regulated, green indicates down-regulated, and dark blue represents unchanged levels. The central diagonal denotes no change, while outer diagonals mark fold-regulation thresholds. miRNAs positioned beyond the outer lines in the upper left or lower right quadrants represent more than threshold up-regulation or down-regulation, respectively.

To characterize the impact of HPV infection on host miRNA expression, we profiled HPV16-positive (SiHa), HPV18-positive (HeLa), and HPV-negative (C33A) cervical cancer cell lines using a cancer-focused miRNA panel. A total of 42 miRNAs were found to be differentially expressed in HPV-infected cells compared to HPV-negative C33A cells. In HPV16-positive SiHa cells, 3 miRNAs were up-regulated, and 39 were down-regulated, whereas HPV18-positive HeLa cells showed 24 up-regulated and 18 down-regulated miRNAs. These findings indicate predominant suppression of miRNA-mediated regulation in HPV16, whereas HPV18 infection showed mixed up-regulation and repression, suggesting subtype-specific effects on miRNA expression. Visualization using a heatmap revealed distinct expression clustering, with altered expression intensity ranging from green (down-regulated) to red (up-regulated), highlighting clear differences between HPV-positive and HPV-negative groups (Fig. 1A). This pattern was further supported by scatterplot representation (Fig. 1B and C), in which miRNAs beyond the fold-change threshold indicate significant dysregulation. In these plots, data points are colored red and green to represent up-regulation and down-regulation, respectively, providing a clear visual contrast of the expression shifts relative to the control group. This analysis further points to the conclusion that high-risk HPV genotypes utilize divergent molecular strategies to remodel host pathways during cervical carcinogenesis. A complete list of dysregulated miRNAs is provided in Table 1. Collectively, these observations demonstrate that HPV16 and HPV18 infections may remodel host posttranscriptional regulatory networks differently, potentially shaping subtype-specific oncogenic pathways during cervical carcinogenesis.

**Table 1.** List of 42 dysregulated miRNA in HPV16- and HPV18-positive cell lines with respect to HPV-negative cell line

Symbol	Up-down regulation (comparing to control group—C33A cell line)	
	Group 1 (SiHa—HPV16)	Group 2 (HeLa—HPV18)
	Fold regulation	Fold regulation
hsa-let-7b-5p	4.03	6.94
hsa-let-7f-5p	−3.99	8.03
hsa-miR-103a-3p	−15.31	−2.09
hsa-miR-106a-5p	−12.49	−3.11
hsa-miR-106b-5p	−2.58	8.59
hsa-miR-107	−12.69	−2.25
hsa-miR-125b-5p	−87.87	−26.81
hsa-miR-132-3p	−21.52	3.27
hsa-miR-26a-5p	−7.91	−3.99
hsa-miR-17-5p	−11.65	−3.10
hsa-miR-181b-5p	2.81	10.49
hsa-miR-27a-3p	−2.01	2.27
hsa-miR-186-5p	−3.43	9.30
hsa-miR-18a-5p	−10.78	6.92
hsa-miR-191-5p	−28.79	−9.77
hsa-miR-192-5p	−4.23	3.46
hsa-miR-148a-3p	−3.29	18.84
hsa-miR-194-5p	−5.01	4.84
hsa-miR-195-5p	−27.51	10.36
hsa-miR-196a-5p	−34.28	−2.22
hsa-miR-19a-3p	−3.11	−2.86
hsa-miR-19b-3p	−4.09	−2.57
hsa-miR-20b-5p	−16.99	16.78
hsa-miR-210-3p	−9.34	3.04
hsa-miR-214-3p	−3.60	6.36
hsa-miR-215-5p	−5.61	5.17
hsa-miR-221-3p	−13.39	−5.35
hsa-miR-222-3p	−25.10	−4.83
hsa-miR-23a-3p	−3.97	2.67
hsa-miR-23b-3p	−5.97	2.58
hsa-miR-24-3p	−3.89	2.81
hsa-miR-25-3p	−10.40	−3.87
hsa-miR-26b-5p	−32.39	2.97
hsa-miR-29b-3p	−3.35	8.42
hsa-miR-29c-3p	−5.18	−2.35
hsa-miR-30c-5p	−16.26	−5.35
hsa-miR-30d-5p	−10.24	3.70
hsa-miR-34a-5p	10.12	100.53
hsa-miR-423-5p	−2.16	5.26
hsa-miR-92b-3p	−5.70	−2.77
hsa-miR-93-5p	−12.42	−3.98
hsa-miR-99a-5p	−363.42	−20.72

### TaqMan-based real-time PCR confirms significant down-regulation of selected miRNAs in HPV-positive cell lines and HPV-transfected models

**Fig. 2.**
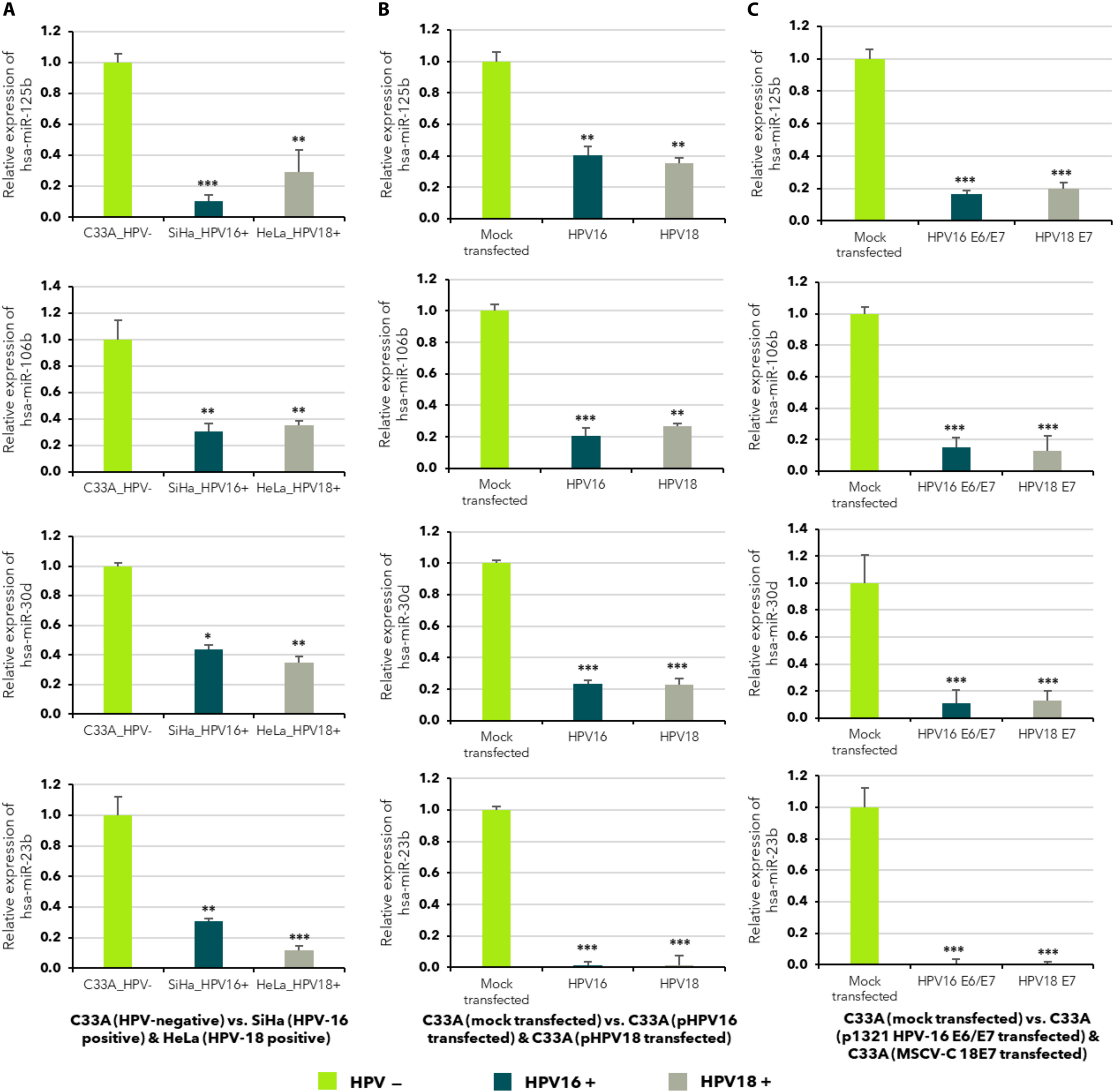
Validation of differentially expressed miRNAs in HPV-associated cervical cancer cell lines and transfection models. Relative expression levels of hsa-miR-125b, hsa-miR-106b, hsa-miR-30d, and hsa-miR-23b were validated using qRT-PCR in HPV-negative and HPV-positive cervical cancer cells, as well as HPV genome and oncogene transfection models. (A) C33A (HPV-negative) cells were used as the reference and set to a fold change of 1. Expression levels in SiHa (HPV16-positive) and HeLa (HPV18-positive) cells were calculated using the 2^−ΔΔCt^ method, normalized to endogenous control and presented relative to C33A. (B) Expression comparison between mock-transfected C33A cells and C33A cells transfected with full-length HPV16 or HPV18 genomes. (C) Expression comparison between mock-transfected C33A cells and C33A cells transfected with HPV16 E6/E7 or HPV18 E7 oncogenes. Across all conditions, consistent down-regulation of the 4 miRNAs was observed in HPV-positive models, with variable reduction patterns between cell lines and transfection types. The results shown as the means of at least 3 experimental replicates plus the standard deviations were calculated and represented as the error bar. *P ≤ 0.05, **P ≤ 0.01, ***P < 0.001. These findings corroborate miRNA suppression during HPV infection and support their involvement in HPV-associated cervical carcinogenesis.

To validate microarray findings and determine whether the identified miRNA signatures are a direct consequence of viral oncoprotein expression, we used a transient transfection model in C33A cells. While we recognize that these short-term assays do not capture the long-term epigenetic landscape of a persistent infection, they provide a high-resolution snapshot of how the host’s regulatory mechanism responds to the immediate presence of E6 and E7. Four miRNAs with strong dysregulation patterns, hsa-miR-125b-5p, hsa-miR-106b-5p, hsa-miR-23b-3p, and hsa-miR-30d-5p, were selected for quantitative expression analysis using TaqMan-based qRT-PCR. These miRNAs were evaluated in HPV-negative C33A cells, naturally HPV-infected SiHa (HPV16) and HeLa (HPV18) cells (Fig. 2A), as well as C33A cells transiently transfected with either full-length HPV16/HPV18 genomes (Fig. 2B) or viral oncogenes E6/E7 (Fig. 2C). In both naturally infected and transfection models, all 4 miRNAs showed consistent down-regulation in HPV-positive conditions, confirming that HPV presence reduces their expression (Fig. 2). Notably, the degree of down-regulation exhibited similar trends in full-genome and E6/E7-transfected cells, implicating HPV oncogenes as key drivers of miRNA suppression. Although both HPV types suppressed these miRNAs, the broader miRNA expression landscape differed between HPV16 and HPV18 models, consistent with oncogene-specific regulatory effects that may contribute to subtype-dependent downstream transcriptional outcomes. The reproducibility of this down-regulation across HPV types and experimental models reinforces the notion that loss of miRNA-mediated regulation may facilitate viral immune evasion, unchecked proliferation, and survival of infected cells. These results validate microarray-based miRNA expression profiles and further indicate that HPV actively represses miRNAs with known roles in inflammation, apoptosis, immune regulation, and tumor suppression, suggesting their involvement in early events of HPV-associated malignant transformation.

### miRWalk mapping analysis reveals broad gene deregulation linked to target repression by hsa-miR-125b-5p, hsa-miR-106b-5p, hsa-miR-30d-5p, and hsa-miR-23b-3p

**Fig. 3.**
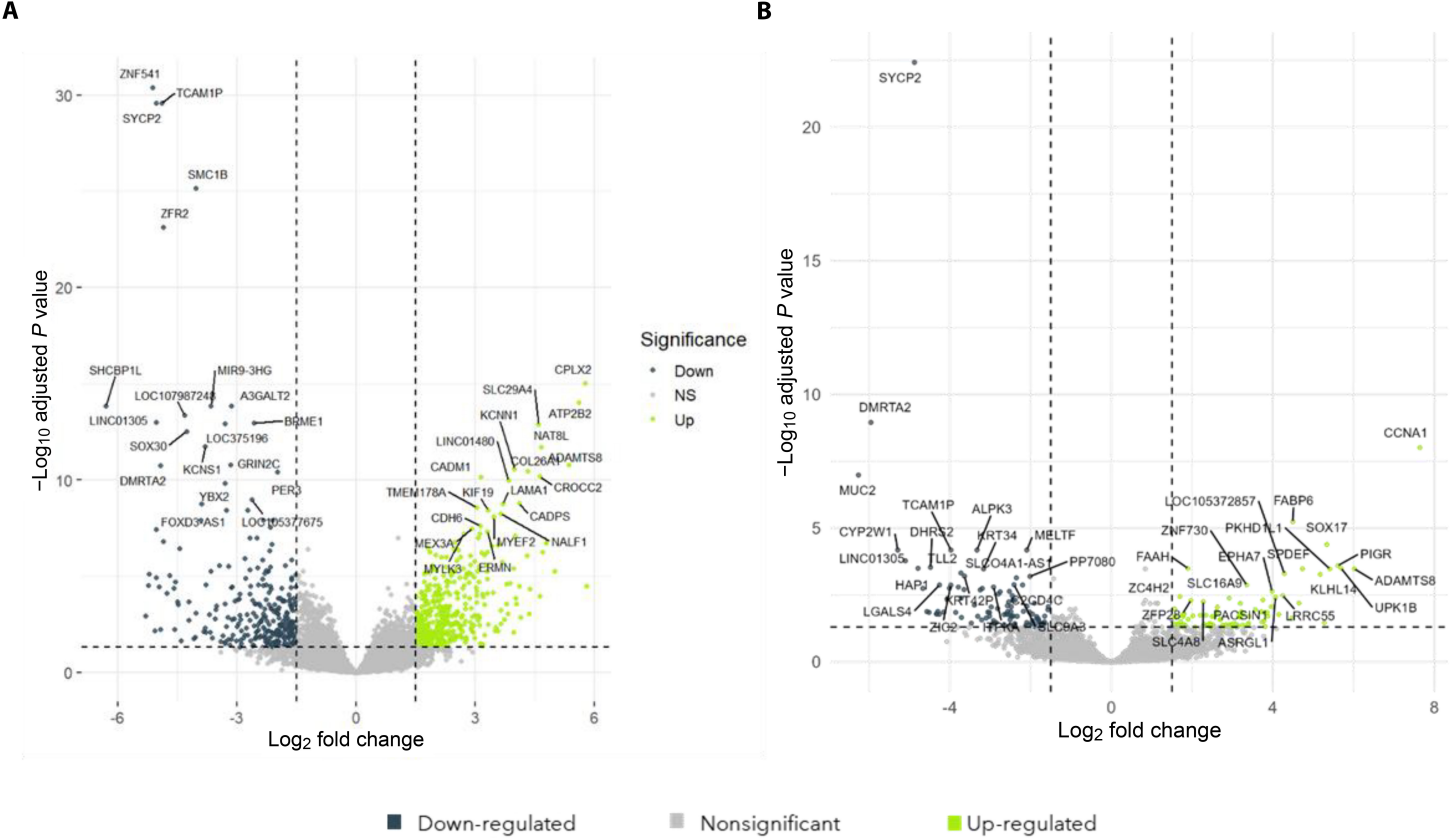
Differential gene expression in HPV-associated cervical cancer. (A) Volcano plot showing gene expression changes between HPV16-positive cervical cancer samples and HPV-negative cervical cancer samples. Differential expression was determined using FDR < 0.05 and log_2_ fold change > 1.5. The top 20 up-regulated and top 20 down-regulated genes are labeled. Light green dots indicate significantly down-regulated genes, dark blue dots indicate significantly up-regulated genes, and gray dots represent nonsignificant genes. (B) Volcano plot showing gene expression changes between HPV18-positive cervical cancer samples and HPV-negative cervical cancer samples using GEO2R differential analysis. Significantly altered genes were defined using FDR < 0.05 and log_2_ fold change > 1.5. The top 20 up-regulated and top 20 down-regulated genes are labeled. Light green dots represent significantly up-regulated genes, dark blue dots represent significantly down-regulated genes, and gray dots denote nonsignificant genes.

To explore the downstream molecular consequences of HPV-associated miRNA suppression, the predicted targets of the 4 validated miRNAs were retrieved from the miRWalk database. Combined analysis identified more than 40,000 predicted target interactions, indicating wide-reaching regulatory effects. Integration of miRWalk target predictions with HPV-associated (HPV-16 and HPV-18 positive) cervical carcinoma expression data (GSE151666) revealed marked transcriptional changes associated with both HPV16 and HPV18 infection (Fig. 3), highlighting distinct HPV-associated gene activation patterns (Table 2). While this initial computational query yielded over 40,000 theoretical interactions reflecting the vast regulatory potential of these miRNAs, we utilized this broad candidate pool only as a starting point for a high-stringency filtration funnel. By integrating these theoretical predictions with HPV-specific cervical carcinoma expression data (GSE151666), we were able to narrow down the initial “computational noise” into a clinically relevant core.

**Table 2.** List of top 20 down-regulated and top 20 up-regulated target genes in HPV-associated cervical cancer

	HPV 16				HPV 18			
S. no.	Down-regulated	Log2FC	Up-regulated	Log2FC	Down-regulated	Log2FC	Up-regulated	Log2FC
1	ZNF541	-5.11917	CPLX2	5.761357	SYCP2	−4.87641	CCNA1	7.643404
2	TCAM1P	−4.89035	ATP2B2	5.6119	DMRTA2	−5.95333	FABP6	4.495198
3	SYCP2	−5.03134	SLC29A4	4.588012	MUC2	−6.26277	SOX17	5.336552
4	SMC1B	−4.03137	NAT8L	4.654842	MELTF	−2.09461	PIGR	5.610982
5	ZFR2	−4.85328	ADAMTS8	5.364396	CYP2W1	−5.28933	FAAH	1.899389
6	A3GALT2	−3.14524	KCNN1	3.992008	ALPK3	−3.32726	UPK1B	5.693418
7	SHCBP1L	−6.29814	COL26A1	4.319604	TCAM1P	−3.9733	SPDEF	4.731976
8	MIR9-3HG	−3.64825	CROCC2	4.634825	LINC01305	−5.10145	PKHD1L1	5.410337
9	LOC107987248	−4.32045	CADM1	3.137845	DHRS2	−4.47868	ADAMTS8	6.01984
10	LINC01305	−5.02064	LINC01480	3.835541	HAP1	−4.78824	LOC105372857	4.286356
11	BRME1	−2.56285	CADPS	4.112199	KRT34	−3.16285	KLHL14	5.17371
12	LOC375196	−3.29152	LAMA1	3.703468	TLL2	−3.72434	ZNF730	3.351038
13	SOX30	−4.26928	TMEM178A	3.03841	KRT42P	−3.63819	LRRC55	4.250092
14	KCNS1	−3.7978	KIF19	3.323217	PP7080	−2.01635	ZC4H2	1.699532
15	GRIN2C	−3.15413	NALF1	3.646526	SLCO4A1-AS1	−2.35213	ASRGL1	4.067063
16	DMRTA2	−4.91889	MYEF2	3.458992	SLC9A3	−2.40892	SLC16A9	2.918805
17	PER3	−1.9747	CDH6	3.120631	C2CD4C	−2.19685	PACSIN1	3.755281
18	YBX2	−3.30207	MEX3A	2.918772	LGALS4	−4.2559	ZFP28	1.969211
19	LOC105377675	−2.61809	ERMN	3.310542	ZIC2	−3.9746	SLC4A8	2.278013
20	FOXD3-AS1	−3.89257	MYLK3	2.725348	ITPKA	−2.91573	EPHA7	3.980045

**Fig. 4.**
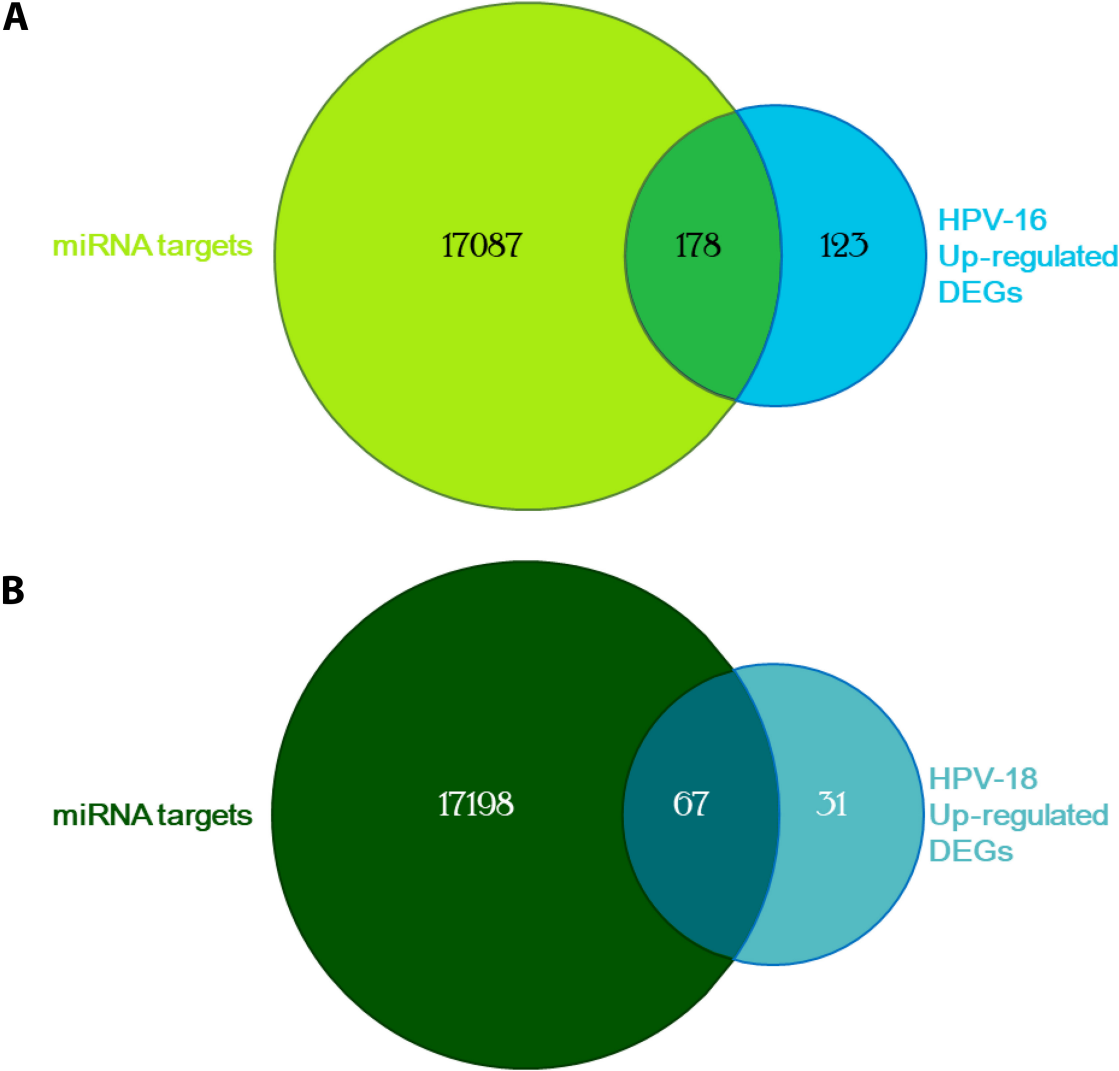
Overlap between miRNA-predicted target genes and up-regulated differentially expressed genes (DEGs) in HPV16- and HPV18-associated cervical cancer. (A) Venn diagram showing the overlap between predicted target genes of down-regulated miRNAs and up-regulated DEGs in HPV16-positive cancer samples. A total of 178 genes overlapped between 17,265 predicted miRNA targets and 301 HPV16 up-regulated DEGs, indicating potential miRNA–mRNA regulatory interactions associated with HPV16 infection. (B) Venn diagram showing the overlap between predicted target genes of down-regulated miRNAs and up-regulated DEGs in HPV18-positive cancer samples. A total of 67 genes overlapped between 17,265 predicted miRNA targets and 98 HPV18 up-regulated DEGs, representing putative miRNA–mRNA regulatory networks in HPV18 infection. These intersections identify candidate genes for downstream enrichment analyses and provide insights into subtype-specific regulatory mechanisms in HPV-associated cervical cancer.

After target prediction and GEO data integration, a total of 17,265 unique miRNA targets were identified for HPV16, and 301 genes were found up-regulated in the HPV16 cervical carcinoma dataset. Among these, 178 genes overlapped between predicted miRNA targets and HPV16-associated up-regulated DEGs, indicating strong enrichment of miRNA-regulated gene activation. For HPV18-positive carcinomas, 17,265 predicted miRNA target genes were compared with 98 up-regulated DEGs, yielding 67 overlapping genes that form putative miRNA–mRNA regulatory networks unique to HPV18 (Fig. 4 and Supplementary Data). These results indicate that a substantial component of HPV-associated gene up-regulation arises from loss of miRNA-mediated repression, particularly in HPV16 infection. In HPV18-positive carcinomas, where both miRNA up-regulation and down-regulation were observed, this derepression-focused analysis captures a dominant regulatory axis while acknowledging the presence of additional counter-regulatory miRNA-mediated suppression.

Notably, many overlapping genes encode TFs, epigenetic modifiers, metabolic regulators, and ECM-associated proteins, suggesting that miRNA repression may act as a molecular trigger that reprograms diverse pathways essential for malignant transformation. These overlapping gene sets were selected for downstream PPI, TF mapping, and pathway enrichment analyses to delineate HPV subtype-specific regulatory circuits and potential therapeutic targets. Accordingly, subsequent functional enrichment analyses reflect regulatory programs mediated by miRNA derepression, rather than the full spectrum of bidirectional miRNA control operative in HPV18-associated tumors.

### GO analysis reveals enriched MFs associated with HPV16- and HPV18-associated miRNA dysregulation

**Fig. 5.**
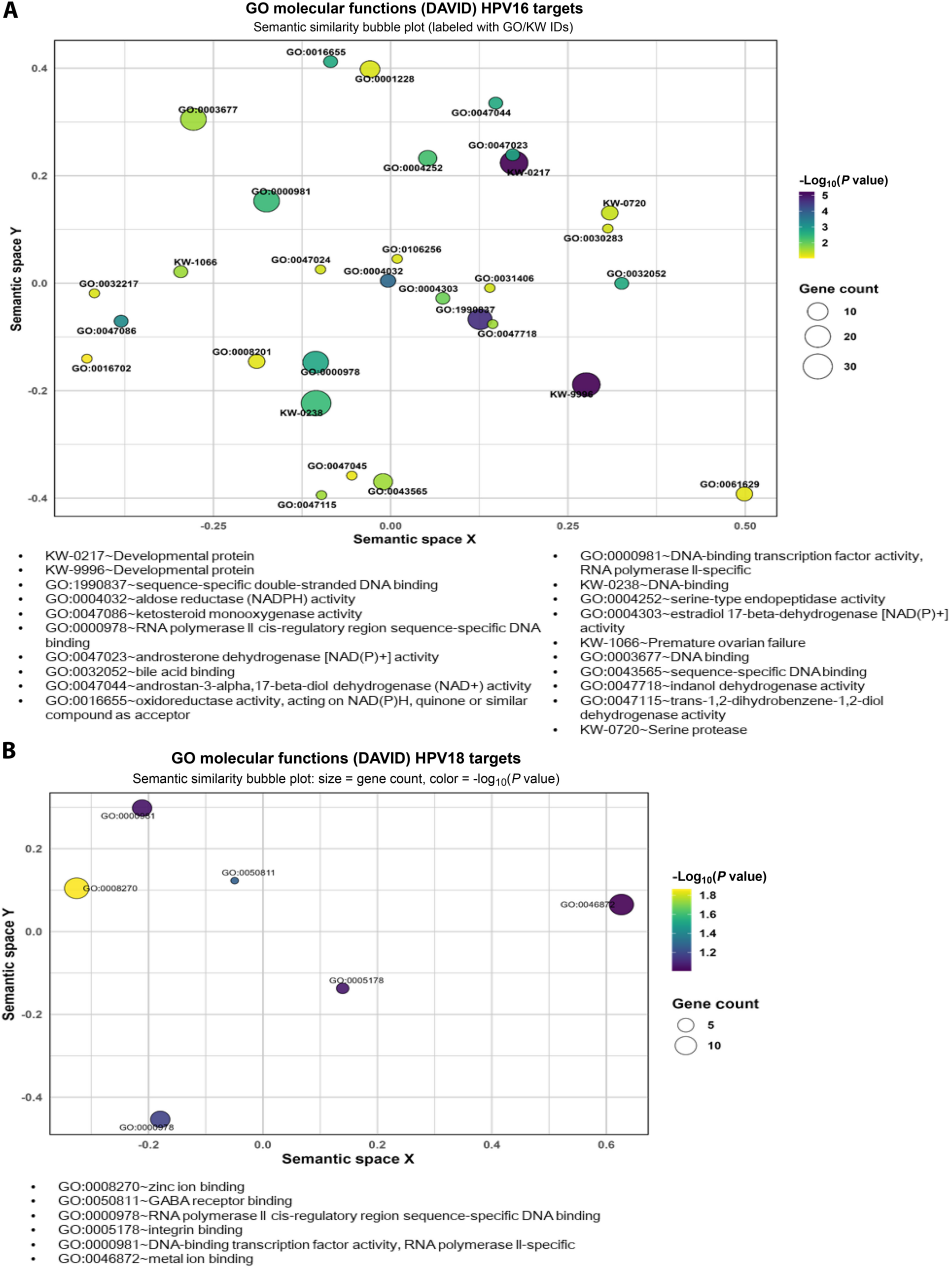
Gene Ontology (GO) molecular function (MF) enrichment of HPV16 and HPV18 target genes visualized using semantic similarity bubble plots. (A) Bubble plot showing enriched GO MF terms and UniProt keywords (KW) associated with HPV16 target genes from DAVID analysis. Bubble size corresponds to the number of genes (Count), and color intensity reflects statistical significance (−log_10_ *P* value). Highly enriched terms include GO:1990837 (sequence-specific double-stranded DNA binding, *P* = 2.15e−5) and GO:0000978 (RNA polymerase II core promoter proximal region sequence-specific DNA binding, *P* = 1.82e−3), indicating strong transcriptional regulation in HPV16 infection. Additional enrichment was observed for oxidoreductase-related activities (GO:0047086, GO:0004032, GO:0047023) and transcription-associated functions (GO:0045944, GO:0003700). The UniProt keyword KW-0217 (developmental protein, *P* = 5.92e−6) further suggests modulation of developmental pathways in HPV16-infected cervical cells. (B) Bubble plot showing enriched GO MF terms associated with HPV18 target genes. Prominent terms included GO:0008270 (zinc ion binding, *P* = 0.0137) and GO:0046872 (metal ion binding, *P* = 0.0987), reflecting metal-binding regulatory interactions linked to HPV18 pathogenesis. Transcription-related categories, such as GO:0000978 (RNA polymerase II cis-regulatory region sequence-specific DNA binding, *P* = 0.0670) and GO:0000981 (DNA-binding transcription factor activity, RNA polymerase II-specific, *P* = 0.0888), indicate active modulation of gene expression. Enrichment of GO:0005178 (integrin binding, *P* = 0.0864) and GO:0050811 (GABA receptor binding, *P* = 0.0538) suggests roles in adhesion-related signaling and ligand-mediated interactions. In both plots, spatial clustering across the axes represents semantic similarity, enabling visualization of functionally related regulatory themes influenced by HPV subtype-specific host–virus interactions.

To characterize the molecular roles associated with dysregulated miRNA target genes, GO MF enrichment and UniProt keyword analyses were performed. A semantic similarity bubble plot was generated to highlight the top-enriched terms in HPV16 and HPV18 infection models (Fig. 5A and B).

In HPV16-associated target genes, strong enrichment of transcription-related functions was observed. Notably, sequence-specific double-stranded DNA binding (GO:1990837; *P* = 2.15 × 10^−5^) and RNA polymerase II core promoter proximal region sequence-specific DNA binding (GO:0000978; *P* = 1.82 × 10^−3^) were significantly enriched, suggesting that HPV16 strongly perturbs host transcriptional machinery. This aligns with the known actions of HPV16 E6/E7 proteins, which hijack transcriptional regulators and co-activators to establish sustained oncogenic gene expression programs [33]. In addition, oxidoreductase activity-associated MFs (GO:0047086, GO:0004032, GO:0047023) were significantly enriched, implying altered redox homeostasis. The enrichment of developmental protein (UniProt KW-0217; *P* = 5.92 × 10^−6^) indicates deregulation of genes associated with cellular differentiation and embryonic pathways, which may be reactivated during HPV-associated malignant progression (Fig. 5A).

In contrast, HPV18-associated target genes displayed a distinct MF enrichment profile. Significant enrichment of zinc ion binding (GO:0008270; *P* = 0.0137) and metal ion binding (GO:0046872; *P* = 0.0987) reflects dependence on zinc-coordinated protein complexes, consistent with HPV18 E7 zinc-binding motifs that modulate chromatin access and TF recruitment [34]. Furthermore, metal ion binding (GO:0046872; *P* = 0.0987) emerged as a suggestive trend. Alongside these functions, 2 transcriptionally relevant categories showed marginal, nonsignificant enrichment: RNA polymerase II cis-regulatory region sequence-specific DNA binding (GO:0000978; *P* = 0.0670) and DNA-binding TF activity, RNA polymerase II-specific (GO:0000981; *P* = 0.0888). While not meeting strict significance thresholds, these suggestive trends hint at potential modulation of host transcriptional programs in HPV18 infection. Similarly, marginal trends were observed for integrin binding (GO:0005178; *P* = 0.0864) and γ-aminobutyric acid (GABA) receptor binding (GO:0050811; *P* = 0.0538). While speculative, these trends may point toward subtype-specific regulation of extracellular signaling and epithelial microenvironmental communication (Fig. 5B). Although GABA signaling is predominantly neuronal, its emerging association with epithelial immune modulation highlights a potential mechanism through which HPV18 may influence microenvironmental interactions. Although these enriched terms approached the conventional significance threshold, they were retained for analysis due to their strong biological relevance and topological consistency with our PPI network. The identification of these “suggestive” enrichment terms is particularly noteworthy, as they represent conserved regulatory programs, such as extracellular signaling and transcriptional control, that were also identified in the high-powered HPV16 model.

Collectively, these results indicate that while both HPV16 and HPV18 manipulate host transcriptional programs, they do so via distinct MF networks. HPV16 appears to influence transcription, metabolic, and developmental pathways, whereas HPV18 targets MFs linked to metalloproteinase and adhesion. These subtype-specific signatures illustrate divergent host–virus regulatory dynamics that may be exploitable for precision therapeutic targeting.

### Pathway enrichment analysis reveals major dysregulated pathways associated with HPV16 and HPV18 infections in cervical cancer

**Fig. 6.**
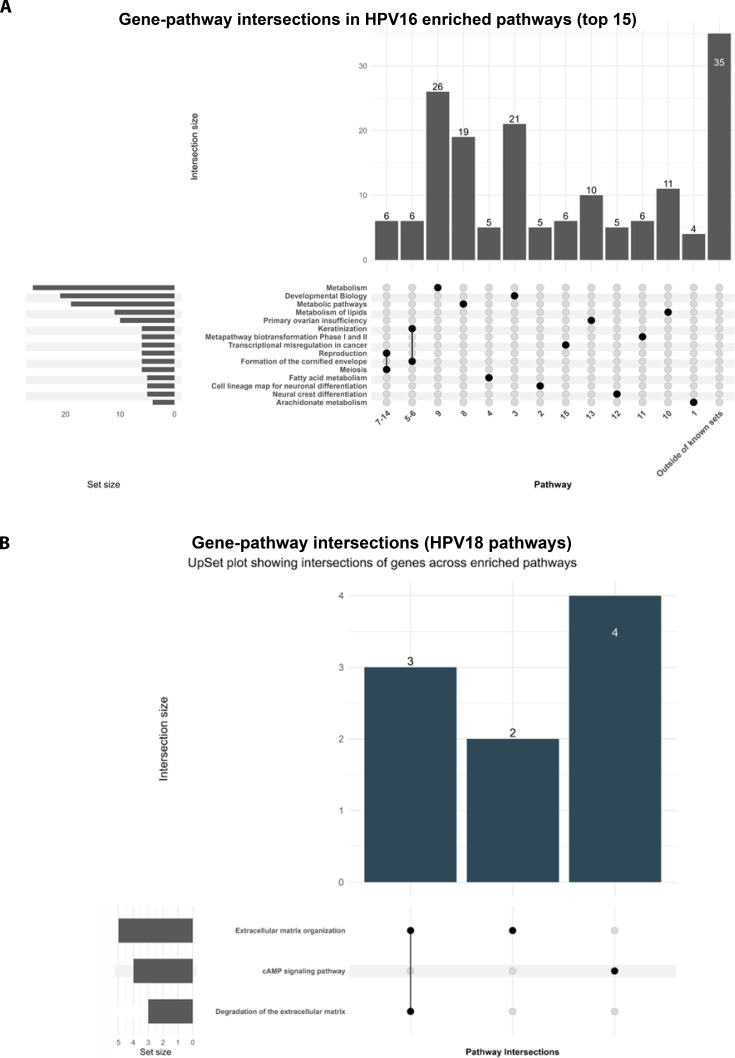
Pathway intersections of HPV16 and HPV18 target genes visualized using UpSet plots. UpSet plots illustrate the intersections among enriched biological pathways derived from DAVID analysis of target genes associated with (A) HPV16 and (B) HPV18 infection. Each vertical bar indicates the number of genes shared across pathway combinations, with connected dots marking the specific intersecting pathways. Horizontal bars represent the total number of genes associated with each individual pathway. (A) HPV16: The top 15 enriched pathways are shown, including Metabolism (*P* = 0.0523), Developmental Biology (*P* = 0.0145), Metabolic pathways (*P* = 0.0712), Meiosis (*P* = 0.0034), and Formation of the cornified envelope (*P* = 0.0044). Notable intersections include genes involved in bile acid and steroid hormone biosynthesis (AKR1C1, AKR1C3, AKR1C2), keratinization, and reproduction-related processes, indicating shared regulatory and metabolic mechanisms influenced by HPV16 in cervical cells. (B) HPV18: Key enriched pathways include Extracellular matrix organization (*P* = 0.0213), cAMP signaling pathway (*P* = 0.0336), and Degradation of the extracellular matrix (*P* = 0.0789). Intersecting genes such as CAPN13, COL26A1, ADAMTS8, and GLI1 highlight remodeling of the ECM and altered signaling dynamics during HPV18 infection. These pathway intersections reveal distinct and overlapping biological networks modulated by HPV16 and HPV18, underscoring subtype-specific metabolic, structural, and signaling alterations that may serve as potential therapeutic targets.

To investigate the biological pathways influenced by dysregulated miRNA targets during HPV-associated cervical carcinogenesis, KEGG, Reactome, and WikiPathways enrichment was performed using DAVID. Pathway significance was assessed using a cutoff of *P* < 0.05, and intersections across HPV16 and HPV18 datasets were visualized using UpSet plots (Fig. 6). These intersections highlight shared and distinct signatures associated with subtype-specific miRNA repression.

In the HPV16-associated dataset, several pathways related to metabolic reprogramming, epithelial differentiation, and transcriptional regulation were highly enriched. Significant pathways included Metabolism (*P* = 0.0523), Developmental Biology (*P* = 0.0145), Metabolic pathways (*P* = 0.0712), Meiosis (*P* = 0.0034), Keratinization (*P* = 0.034), Transcriptional misregulation in cancer (*P* = 0.021), and Formation of the cornified envelope (*P* = 0.0044). Notable gene intersections included those involved in bile acid and steroid hormone biosynthesis (AKR1C1, AKR1C3, and AKR1C2), keratinization (KRT33A, KRT15, KRT24, KRT86, KRT31, and LCE1C), transcriptional misregulation in cancer (NGFR, CDKN2C, TLX3, PAX7, PLAT, and MLF1), cell differentiation (SOX2, TLX3, PAX7, and TLX2), and reproduction-related processes (Fig. 6 and Supplementary Data). These findings suggest that HPV16 may rewire epithelial differentiation and transcriptional machinery, reflecting oncogenic shifts in keratinocyte biology.

In contrast, HPV18-associated targets demonstrated enrichment in pathways governing ECM remodeling and signaling. Major pathways included Extracellular matrix organization (*P* = 0.0213), cAMP signaling pathway (*P* = 0.0336), and Degradation of the extracellular matrix (*P* = 0.0789). These pathways involved genes critical for matrix remodeling and epithelial signaling, such as CAPN13, COL26A1, ADAMTS8, and GLI1, indicating that HPV18 infection may favor alterations in the microenvironment, invasion, adhesion, and signal transduction (Fig. 6 and Supplementary Data). Notably, the enrichment of adenosine 3′,5′-monophosphate (cAMP) signaling, a pathway implicated in innate immune suppression and tumor modulation, aligns with reports highlighting its context-dependent effect in HPV-associated malignancies.

Together, these findings demonstrate that miRNA dysregulation in HPV16 infection predominantly targets metabolic, keratinization, and transcriptional misregulation pathways, whereas HPV18-associated deregulation emphasizes ECM remodeling and signaling perturbation (particularly cAMP-mediated pathways). These subtype-specific molecular footprints suggest distinct oncogenic strategies driven by miRNA–mRNA networks and highlight potential therapeutic nodes that may be selectively targeted in HPV16- and HPV18-associated cervical cancer.

### TF–gene regulatory network analysis identifies distinct upstream regulators driving HPV16- and HPV18-mediated gene expression programs

**Fig. 7.**
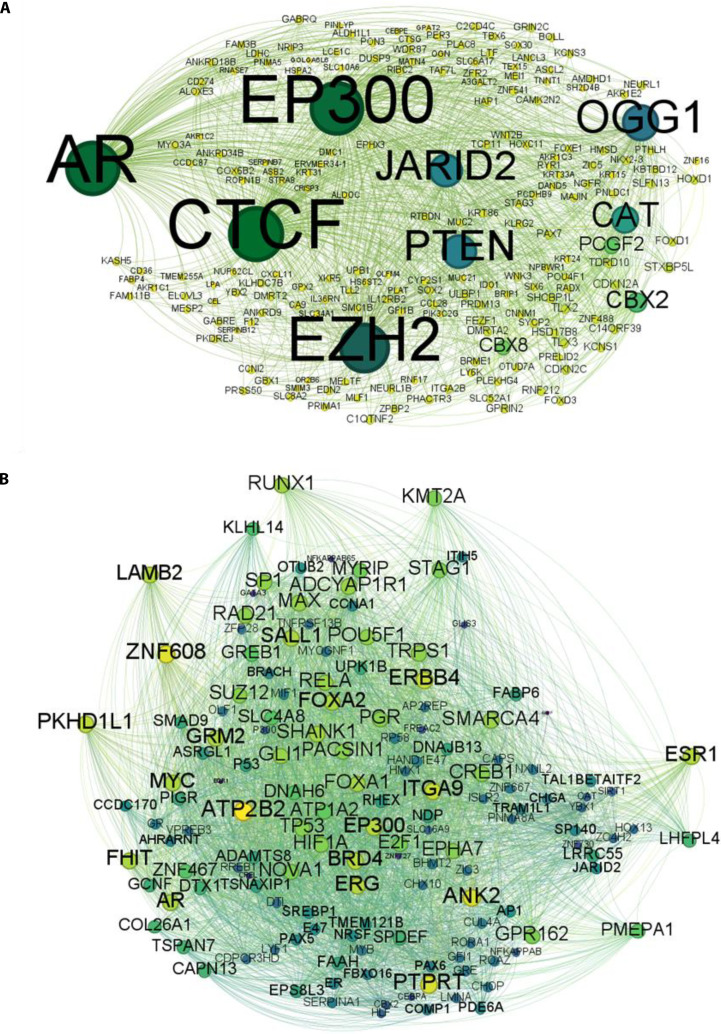
Transcription factors (TFs) and their associated gene targets in HPV-associated cervical cancer. (A) Network of TFs and associated gene targets up-regulated in HPV16-associated cervical carcinoma. TFs were identified through DAVID enrichment of predicted targets of down-regulated miRNAs (hsa-miR-23b, hsa-miR-30d, hsa-miR-106b, and hsa-miR-125b). Key TF hubs, including JARID2, EP300, OGG1, and CBX2, are shown interacting with multiple gene targets, reflecting their regulatory influence in HPV16-driven carcinogenesis. (B) Network of TFs and associated gene targets up-regulated in HPV18-associated cervical carcinoma, derived from DAVID enrichment using predicted targets of the same dysregulated miRNAs. Prominent TF hubs include GCNF, CBX2, ERG, CUL4A, and JARID2, demonstrating both distinct and overlapping transcriptional regulatory patterns in HPV18 infection compared to HPV16. In both networks, nodes represent TFs and gene targets, edges denote TF–gene associations, and node size represents degree of connectivity, indicating their regulatory impact within the network.

To determine the regulatory hierarchy governing dysregulated gene expression in HPV-associated cervical cancer, TF enrichment was analyzed for overlapping miRNA target genes in the HPV16 and HPV18 datasets. TF associations were identified through DAVID-based enrichment and visualized to illustrate subtype-specific regulatory hubs that modulate gene expression (Fig. 7).

In HPV16-associated networks, TFs involved in cellular differentiation and oncogenic reprogramming were prominently enriched. Key TF associations included JARID2, CAT, EP300, OGG1, EZH2, PTEN, and CBX2. The altered expression of these genes underscores their combined influence on cervical cancer pathogenesis through transcriptional misregulation. Other important genes, such as SOX2, PAX5, PAX7, and KAT6A, which are known to influence stemness, chromatin remodeling, and lineage specification, were enriched. Their enrichment indicates that HPV16 may promote transformation through dedifferentiation-driven transcriptional plasticity, consistent with observed keratinization and developmental pathway dysregulation (Fig. 7A and Supplementary Data—Sheet 5). This aligns with the enriched GO term developmental protein (KW-0217) and the keratinocyte differentiation pathways highlighted in the previous section. The co-occurrence of TFs linked to transcription, epithelial identity, and embryonic signaling underscores the possibility that HPV16 reactivates dormant developmental programs to establish a malignant proliferative state. These genes collectively modulate key developmental and embryonic programs while suppressing normal keratinization pathways, thereby promoting a stem-like, poorly differentiated phenotype characteristic of cervical cancer progression.

Conversely, HPV18-associated TF enrichment revealed a predominantly extracellular signaling and stress-response regulatory landscape. Notable TF associations included GLI1, SPIB, KLF10, and KLF11, many of which are linked to Hedgehog signaling, lipid metabolism, immune modulation, and stromal communication. Other TFs include GCNF (germ cell nuclear factor), CBX2, ERG, ANK2, LAMB2, CUL4A, and JARID2. It is interesting to note that both CBX2 and JARID2 were shared across both HPV types; however, their regulatory partners differed, suggesting context-specific roles in gene regulation. The presence of ERG, a member of the ETS TF family, and CUL4A, an E3 ubiquitin ligase involved in chromatin remodeling and DNA repair, underscores the role of transcriptional and posttranslational regulation in HPV18-mediated cancer progression. These TFs correlate with ECM remodeling pathways and the cAMP signaling axis, consistent with the pathway enrichment results described in the previous section. This suggests that HPV18 may influence transformation through microenvironment-driven signaling feedback loops rather than differentiation reprogramming. In particular, the enrichment of GLI1, a key Hedgehog effector linked to invasive phenotypes, may reflect a regulatory mechanism that supports tissue invasion and immune evasion in HPV18-driven tumors (Fig. 7B). Collectively, the enrichment of GLI1, SPIB, KLF10, KLF11, GCNF, CBX2, ERG, ANK2, LAMB2, CUL4A, and JARID2 points toward oncogenesis by reactivating embryonic programs, enforcing epigenetic repression, disrupting cell–matrix interactions, remodeling the ECM, and removing key differentiation and growth-inhibitory pathways.

Together, these TF–gene network signatures demonstrate that HPV16 infection predominantly rewires regulatory circuits governing proliferation and differentiation, whereas HPV18 infection preferentially activates transcriptional programs associated with extracellular signaling, stromal modification, and immune modulatory pathways. These subtype-specific TF regulatory nodes may serve as candidate biomarkers or intervention points for precision targeting in HPV16- versus HPV18-associated cervical cancers.

### PPI network topology reveals key hub genes linked to HPV subtype-specific carcinogenic progression

**Fig. 8.**
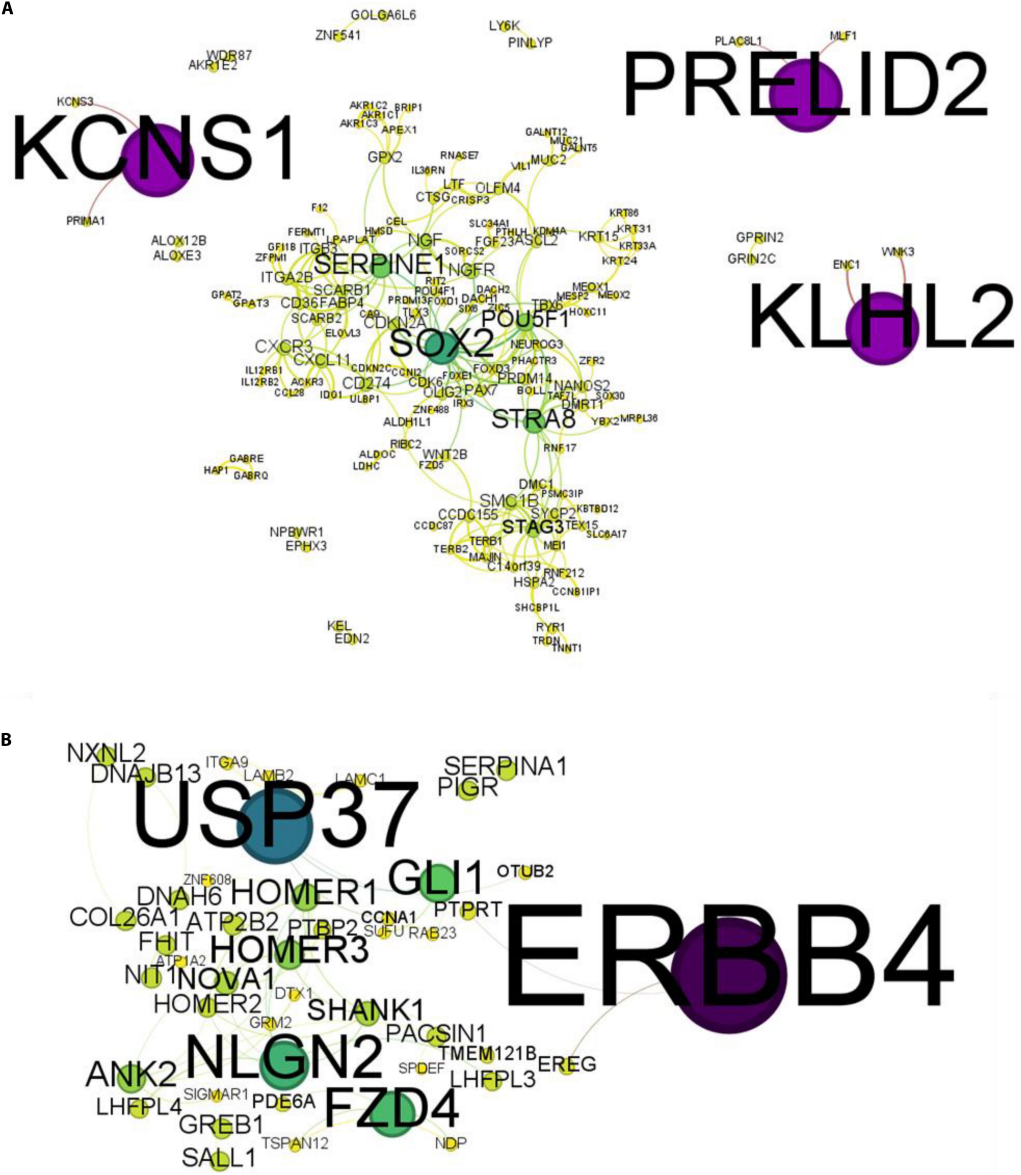
Protein–protein interaction (PPI) networks of prioritized hub genes in HPV-associated cervical cancer. (A) STRING PPI network showing the top 20 prioritized hub genes in HPV16-associated cervical cancer, derived from predicted targets of down-regulated miRNAs (hsa-miR-23b, hsa-miR-30d, hsa-miR-106b, and hsa-miR-125b). Key hubs including PRELID2, KLHL2, KCNS1, SOX2, and SERPINE1 exhibit high connectivity and central network influence, indicating critical roles in HPV16-driven oncogenic regulation. (B) STRING PPI network representing the top 20 prioritized hub genes in HPV18-associated cervical cancer, based on predicted targets of the same dysregulated miRNAs in HPV18 context. Prominent hubs such as ERBB4, USP37, NLGN2, FZD4, and GLI1 display strong interaction patterns, suggesting their potential regulatory impact in HPV18-mediated carcinogenic processes. In both networks, node size corresponds to interaction degree and edges represent protein–protein associations, visually depicting central regulators within HPV subtype-specific molecular networks.

**Fig. 9.**
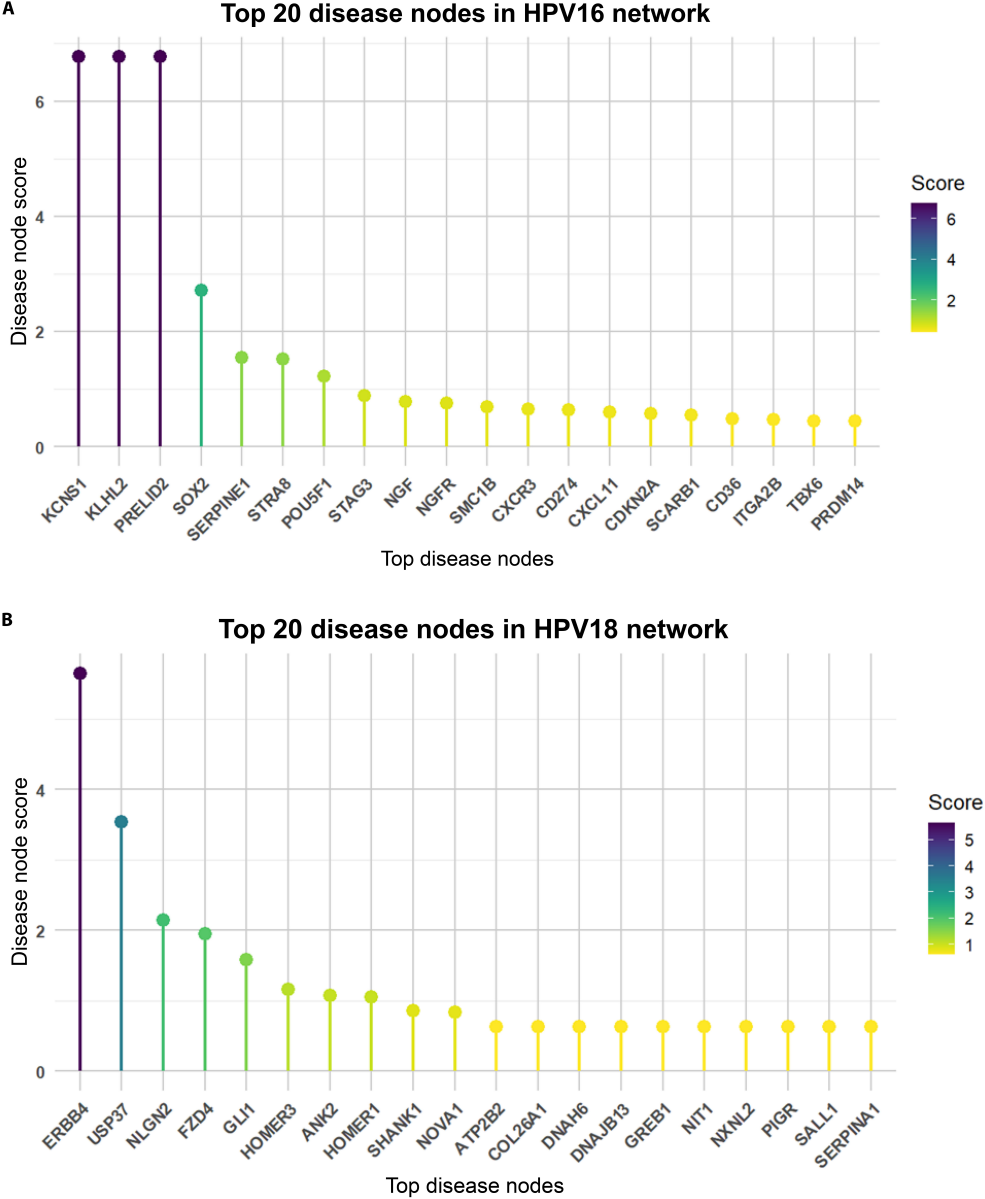
Prioritization of hub genes in HPV-associated cervical cancer using disease node scores. (A) Lollipop plot showing the top 20 prioritized hub genes in HPV16-associated cervical cancer, based on disease node scores calculated from STRING PPI data for predicted targets of down-regulated miRNAs (hsa-miR-23b, hsa-miR-30d, hsa-miR-106b, and hsa-miR-125b). Highly ranked hubs such as PRELID2, KLHL2, KCNS1, SOX2, and SERPINE1 exhibit strong centrality, indicating key regulatory influence in HPV16-driven carcinogenesis. (B) Lollipop plot showing the top 20 prioritized hub genes in HPV18-associated cervical cancer, derived using disease node scores for predicted targets of the same dysregulated miRNAs in HPV18 context. Prominent nodes including ERBB4, USP37, NLGN2, FZD4, and GLI1 demonstrate notable centrality, suggesting important regulatory roles in HPV18-mediated pathways. In both plots, each lollipop represents a gene, with stem height reflecting its disease node score. This visualization highlights high-confidence target genes for functional validation and potential biomarker or therapeutic exploration in HPV subtype-specific cervical cancer.

To identify key regulatory genes occupying topologically significant positions associated with miRNA-mediated disease interactions, PPI analysis was performed using the targets of dysregulated miRNAs. Differentially expressed target genes were retrieved from STRING and imported into Cytoscape, and network analysis was performed in RStudio to obtain a robust interaction profile (Fig. 8). Disease node scores, derived from STRING PPI network statistics, were used to rank hub genes by network centrality and influence. A visual representation of these rankings was generated using a lollipop plot (Fig. 9), and the complete list of top 20 hub genes for both HPV16 and HPV18 networks is provided in Supplementary Data.

Within the HPV16-associated cervical cancer network, 234 enriched genes were analyzed, from which the top 20 hub genes were identified. These included PRELID2, KLHL2, KCNS1, SOX2, and SERPINE1 (Table 3). These genes exhibited elevated disease node scores, indicating strong connectivity and regulatory potential. Among them, SOX2 (a transcriptional regulator associated with stemness and dedifferentiation) and SERPINE1 (an ECM remodeling factor) reaffirm established biological roles in HPV16-driven oncogenesis. Meanwhile, PRELID2 and KCNS1, although less characterized in cervical cancer, are functionally linked to metabolism and membrane signaling, suggesting previously unexplored regulatory elements that may warrant further investigation. These nodes may serve as putative regulatory bottlenecks linking metabolism and membrane signaling to the broader oncogenic program.

**Table 3.** List of top 20 disease node hub genes in HPV-associated cervical cancer

HPV16			HPV18		
Gene name	Degree	DiseaseNodeScore	Gene name	Degree	DiseaseNodeScore
PRELID2	2	6.765745008	ERBB4	2	5.642857143
KLHL2	2	6.765745008	USP37	3	3.537981859
KCNS1	2	6.765745008	NLGN2	7	2.140333069
SOX2	21	2.708300223	FZD4	3	1.951664876
SERPINE1	15	1.546156754	GLI1	3	1.576788024
STRA8	15	1.515038236	HOMER3	6	1.159039242
POU5F1	14	1.218301885	ANK2	5	1.069400104
STAG3	14	0.880165846	HOMER1	6	1.045078441
NGF	9	0.780618837	SHANK1	5	0.854232403
NGFR	7	0.752193782	NOVA1	2	0.83179371
SMC1B	11	0.688466347	SALL1	1	0.626984127
CXCR3	10	0.650291634	GREB1	1	0.626984127
CD274	8	0.630278591	COL26A1	1	0.626984127
CXCL11	9	0.601383851	NXNL2	1	0.626984127
CDKN2A	8	0.575637391	DNAJB13	1	0.626984127
SCARB1	8	0.547360642	DNAH6	1	0.626984127
CD36	7	0.479502457	PIGR	1	0.626984127
ITGA2B	8	0.463734064	SERPINA1	1	0.626984127
TBX6	6	0.447250789	ATP2B2	2	0.626984127
PRDM14	7	0.442263845	NIT1	1	0.626984127

Similarly, analysis of the HPV18-associated network comprising 89 enriched genes identified the top 20 hub genes, including ERBB4, USP37, NLGN2, FZD4, and GLI1 (Table 3). ERBB4, a receptor tyrosine kinase of the epidermal growth factor receptor (EGFR) family, and FZD4, a Wnt signaling receptor, highlight the involvement of canonical oncogenic signaling pathways within the HPV18-associated regulatory network. Identification of GLI1, a transcriptional effector of the Hedgehog pathway, further supports the topological involvement of developmental and morphogenetic signaling circuits at the network level during HPV18-mediated transformation. Collectively, the lollipop plots (Fig. 9) visually capture these influential hub nodes as high-priority targets, providing a ranked framework for prioritizing regulatory genes as potential biomarkers or therapeutic targets unique to HPV subtype-specific cervical cancer progression (Tables 4 and 5).

**Table 4.** Topological and functional characterization of HPV16 network genes

S. no.	Category	Gene symbol	Network role	Functional significance in malignancy	References
1	Novel putative	PRELID2	Top bottleneck (score: 6.76)	Involved in mitochondrial lipid transport; suggests a novel metabolic vulnerability in HPV16.	[78]
2	Novel putative	KLHL2	Top bottleneck (score: 6.76)	A Cullin-3 substrate adapter for ubiquitination; likely facilitates viral-mediated protein degradation.	[79]
3	Novel putative	KCNS1	Top bottleneck (score: 6.76)	Potassium channel subunit; ion channel dysregulation is an emerging hallmark of tumor progression.	[80]
4	Validated anchor	SOX2	Primary hub (degree: 21)	Master regulator of cancer stem cells (CSCs); drives pluripotency and tumor self-renewal.	[81]
5	Validated anchor	SERPINE1	Major hub (degree: 15)	Promotes extracellular matrix (ECM) remodeling and angiogenesis; critical for metastasis.	[82]
6	Novel putative	STRA8	Major hub (degree: 15)	Involved in retinoic acid-induced differentiation; high degree suggests a role in maintaining the cell differentiation	[78]
7	Validated anchor	POU5F1 (OCT4)	Hub (degree: 14)	Works with SOX2 to maintain stem-like phenotypes and impaired cell proliferation and invasion in cervical cancer.	[83]
8	Novel putative	STAG3	Hub (degree: 14)	Meiosis-specific cohesin; its expression in somatic cells (meiomitosis) promotes genomic instability.	[78]
9	Novel putative	NGF	Hub (degree: 9)	Nerve growth factor; can promote tumor cell proliferation and survival via autocrine loops. Recent studies demonstrates role in cervical cancer progress through interaction with Hippo/YAP pathway	[84,85]
10	Novel putative	NGFR	Score: 0.75	The receptor for NGF; shown to control perineural invasion and metastasis in oral cancer.	[86]
11	Validated hub	SMC1B	Hub (degree: 11)	Cohesin subunit; shown to regulate EMT via PI3K/AKT pathway	[87]
12	Novel putative	CXCR3	Hub (degree: 10)	Chemokine receptor involved in the recruitment of immune cells to the tumor microenvironment.	[78]
13	Validated anchor	CD274 (PD-L1)	Score: 0.63	A key immune checkpoint protein; mediates immune evasion in HPV-positive tumors.	[88]
14	Novel putative	CXCL11	Score: 0.60	Ligand for CXCR3; part of the inflammatory signaling network.	[78]
15	Validated anchor	CDKN2A (p16)	Score: 0.57	The primary clinical diagnostic marker for high-risk HPV; controls the G1/S cell cycle checkpoint.	[78,89]
16	Novel putative	SCARB1	Score: 0.54	Scavenger receptor for high-density lipoproteins; linked to cholesterol-driven proliferation. Act as host cell receptor for virus entry	[78]
17	Validated anchor	CD36	Score: 0.47	Associated with lipid metabolism and metastatic potential; promote EMT and metastasis interacting with TGF-β	[90]
18	Novel putative	ITGA2B	Score: 0.46	Integrin α2b; involved in cell adhesion and interaction with the basement membrane.	[78]
19	Novel putative	TBX6	Score: 0.44	Transcription factor involved in development; members of this family (like TBX3) are known HPV-cooperating partners.	[78,91]
20	Validated anchor	PRDM14	Score: 0.44	Silenced via methylation. Loss of PRDM14 leads to apoptosis evasion and promotes viral-mediated transformation.	[92]

**Table 5.** Topological and functional characterization of HPV18 network genes

S. no.	Category	Gene symbol	Network role	Functional significance in malignancy	References
1	Validated anchor	ERBB4	Key bridge (centrality: 1.0)	Receptor tyrosine kinase in the EGFR family; mediates growth signaling and cell survival.	[93]
2	Novel putative	USP37	Bottleneck (score: 3.53)	A deubiquitinase that stabilizes oncoproteins (like ERK1/2) to promote S-phase entry.	[78]
3	Novel putative	NLGN2	Primary hub (degree: 7)	A cell-adhesion molecule; suggests a role in maintaining the structural architecture of the tumor.	[78]
4	Validated anchor	FZD4	Pathway bridge (score: 1.95)	A receptor for the Wnt signaling pathway; crucial for oncogenic progression and polarity.	[78,94]
5	Validated anchor	GLI1	Pathway bridge (score: 1.57)	The main effector of Hedgehog signaling; drives aggressive growth and stemness.	[95]
6	Novel putative	HOMER3	Hub (degree: 6)	Scaffolding protein that organizes signaling complexes at the plasma membrane.	[78]
7	Novel Putative	ANK2	Hub (degree: 5)	Ankyrin protein; links the cytoskeleton to the membrane to regulate cell shape and motility.	[78]
8	Novel putative	HOMER1	Hub (degree: 6)	Coordinates with HOMER3 to facilitate intracellular calcium signaling.	[78]
9	Novel putative	SHANK1	Hub (degree: 5)	A master scaffolding protein; high clustering suggests it forms a core signaling unit in the network.	[78]
10	Novel putative	NOVA1	Score: 0.83	RNA-binding protein; low expression in viral E6 and E7 oncoproteins decreases NOVA1 expression to manipulate host cell RNA processing	[96]
11	Novel putative	SALL1	Score: 0.62	A Spalt-like transcription factor associated with developmental signaling and stemness. Role in various cancers	[97]
12	Novel putative	GREB1	Score: 0.62	An estrogen-responsive gene; potentially mediates hormonal influence on HPV18 tumors.	[78]
13	Novel putative	COL26A1	Score: 0.62	A collagen component; likely contributes to the rigidity and signaling of the tumor ECM.	[78]
14	Novel putative	NXNL2	Score: 0.62	Nucleoredoxin-like protein; promotes proliferation through regulating AKT pathway	[98]
15	Novel putative	DNAJB13	Score: 0.62	A molecular chaperone (HSP40 family)	[78]
16	Novel putative	DNAH6	Score: 0.62	Dynein motor protein; involved in intracellular transport and potentially cell motility.	[78]
17	Novel putative	PIGR	Score: 0.62	Polymeric immunoglobulin receptor; mediates the transport of antibodies and immune response.	[78,99]
18	Novel putative	SERPINA1	Score: 0.62	A protease inhibitor; involved in immunological regulation in cancer.	[100]
19	Novel putative	ATP2B2	Score: 0.62	A calcium-transporting ATPase; critical for maintaining intracellular calcium homeostasis.	[78]
20	Novel putative	NIT1	Score: 0.62	Nitrilase-like protein; often acts as a tumor suppressor.	[101]

### Differential expression of prioritized disease hub genes reveals significant mRNA alterations and potential biomarker candidates in HPV16 and HPV18 cervical cancers

To validate the regulatory potential of hub genes identified in network analysis, mRNA expression profiling was performed using UALCAN based on TCGA-CESC datasets. A total of 19 prioritized hub genes from HPV16-associated CESC and 17 hub genes from HPV18-associated CESC were evaluated by comparing expression levels between normal cervical tissues (*n* = 3) and primary cervical tumor samples (*n* = 305). This analysis enabled the assessment of subtype-specific differential expression patterns and the identification of potential biomarkers linked to HPV-associated carcinogenesis.

**Fig. 10.**
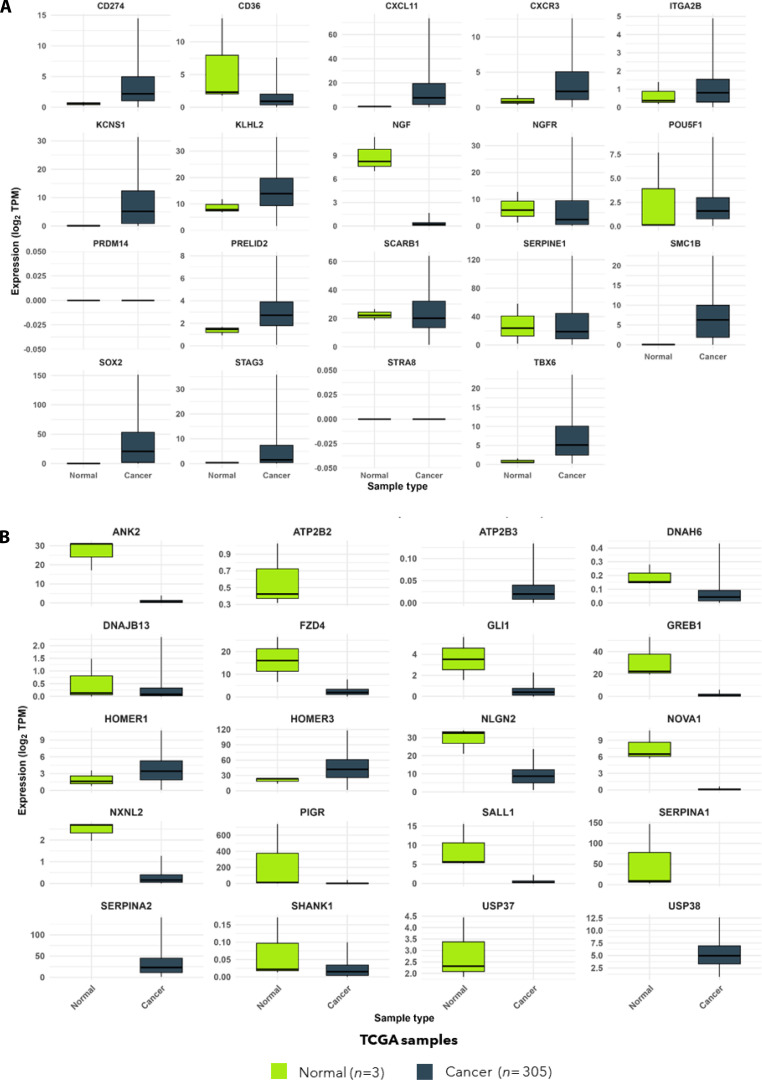
Differential expression of prioritized hub genes in HPV-associated cervical squamous cell carcinoma (CESC). (A) Box plots show the mRNA expression levels of 19 prioritized hub genes in normal cervical tissues (*n* = 3) versus primary tumor samples (*n* = 305) for HPV16-associated CESC, analyzed using the UALCAN TCGA dataset. Hub genes KCNS1, SOX2, PRELID2, SMC1B, STAG3, CXCL11, CD274, CXCR3, and TBX6 were significantly up-regulated in tumors (*P* < 0.05), while NGF was significantly down-regulated. KLHL2, SERPINE1, POU5F1, NGFR, SCARB1, CD36, and ITGA2B showed variable but nonsignificant expression differences, and STRA8 and PRDM14 showed no detectable expression in either group. (B) Box plots show log_2_ TPM values of 17 prioritized hub genes in HPV18-associated CESC, comparing normal cervical tissues (*n* = 3, green, left) and tumor tissues (*n* = 305, dark blue, right). Significant down-regulation was observed for NLGN2, FZD4, GLI1, ANK2, NOVA1, and ATP2B2 (*P* < 0.05), whereas HOMER3 showed significant up-regulation in tumors. USP37, HOMER1, SHANK1, SALL1, GREB1, NXNL2, DNAJB13, DNAH6, PIGR, and SERPINA1 exhibited nonsignificant variation. In both panels, boxes indicate the interquartile range (IQR), central lines show medians, whiskers denote distribution range, and outliers are represented as individual points. These expression trends highlight subtype-specific gene alterations with potential biomarker and therapeutic relevance in HPV-associated cervical cancer.

In HPV16-positive cervical cancer, several hub genes demonstrated significant up-regulation in tumor tissues relative to normal controls. These included KCNS1, SOX2, PRELID2, SMC1B, STAG3, CXCL11, CD274, CXCR3, and TBX6, suggesting their involvement in tumor initiation, growth, and immune modulation. A notable down-regulation of NGF was observed, indicating a possible loss of neurotrophic signaling within tumor tissue. Despite its reduced expression in TCGA tumor samples, NGF emerged as a disease node due to its high interaction centrality within the HPV16-associated regulatory network, suggesting that suppression of key signaling intermediates may itself represent a biologically relevant feature of HPV-associated tumor evolution. Additional genes, such as KLHL2, SERPINE1, POU5F1, NGFR, SCARB1, CD36, and ITGA2B, exhibited variable expression patterns without statistically significant differences, whereas STRA8 and PRDM14 displayed no detectable expression in either the normal or tumor groups. Importantly, 2 genes that are significantly altered, SOX2, a core transcriptional stemness regulator, and CD274 (PD-L1), an immune checkpoint molecule, highlight the interplay between oncogenic reprogramming and immune evasion in HPV16-driven disease progression (Fig. 10).

In HPV18-positive cervical cancer, differential expression analysis identified significant down-regulation of several hub genes, including NLGN2, FZD4, GLI1, ANK2, NOVA1, and ATP2B2 (*P* < 0.05), emphasizing disruption of epithelial signaling, Hedgehog pathway regulation, and synaptic-like adhesion mechanisms in tumor tissues. Notably, several HPV18-associated hub genes identified in the GEO-based network analysis, including GLI1, FZD4, and NLGN2, were significantly down-regulated in TCGA-CESC tumor tissues relative to normal cervix. This apparent directional shift reflects the distinct biological contexts captured by the 2 datasets. While the GEO dataset contrasts HPV18-positive and HPV-negative cervical cancers, highlighting HPV-attributable transcriptional perturbations, TCGA analysis compares established tumors with normal tissue, thereby representing later-stage disease states. The suppression of these hubs in TCGA tumors indicates a stage-specific or context-dependent association. Without direct functional evidence, these down-regulated hubs should be interpreted as topological features of pathway modulation rather than active oncogenic drivers.

Conversely, HOMER3 was significantly up-regulated in tumor samples, potentially reflecting altered calcium signaling or receptor-scaffold interactions that influence cellular communication in HPV18-mediated tumorigenesis. Other prioritized hub genes, including USP37, HOMER1, SHANK1, SALL1, GREB1, NXNL2, DNAJB13, DNAH6, PIGR, and SERPINA1, showed variable trends without significance. These subtype-specific expression profiles (Fig. 10) collectively suggest that HPV16 and HPV18 influence distinct oncogenic pathways, driven, respectively, by transcriptional deregulation and immune modulation (HPV16) versus microenvironmental signaling and scaffold-associated communication (HPV18).

Taken together, these findings indicate that specific hub genes altered during persistent HPV infection may serve as novel molecular biomarkers and potential therapeutic targets. Their differential regulation underscores the contrasting biological strategies adopted by HPV16 and HPV18 in cervical cancer progression.

### Prioritized disease hub genes and associated drug interactions reveal putative clinically actionable targets in HPV16- and HPV18-associated cervical cancer

**Fig. 11.**
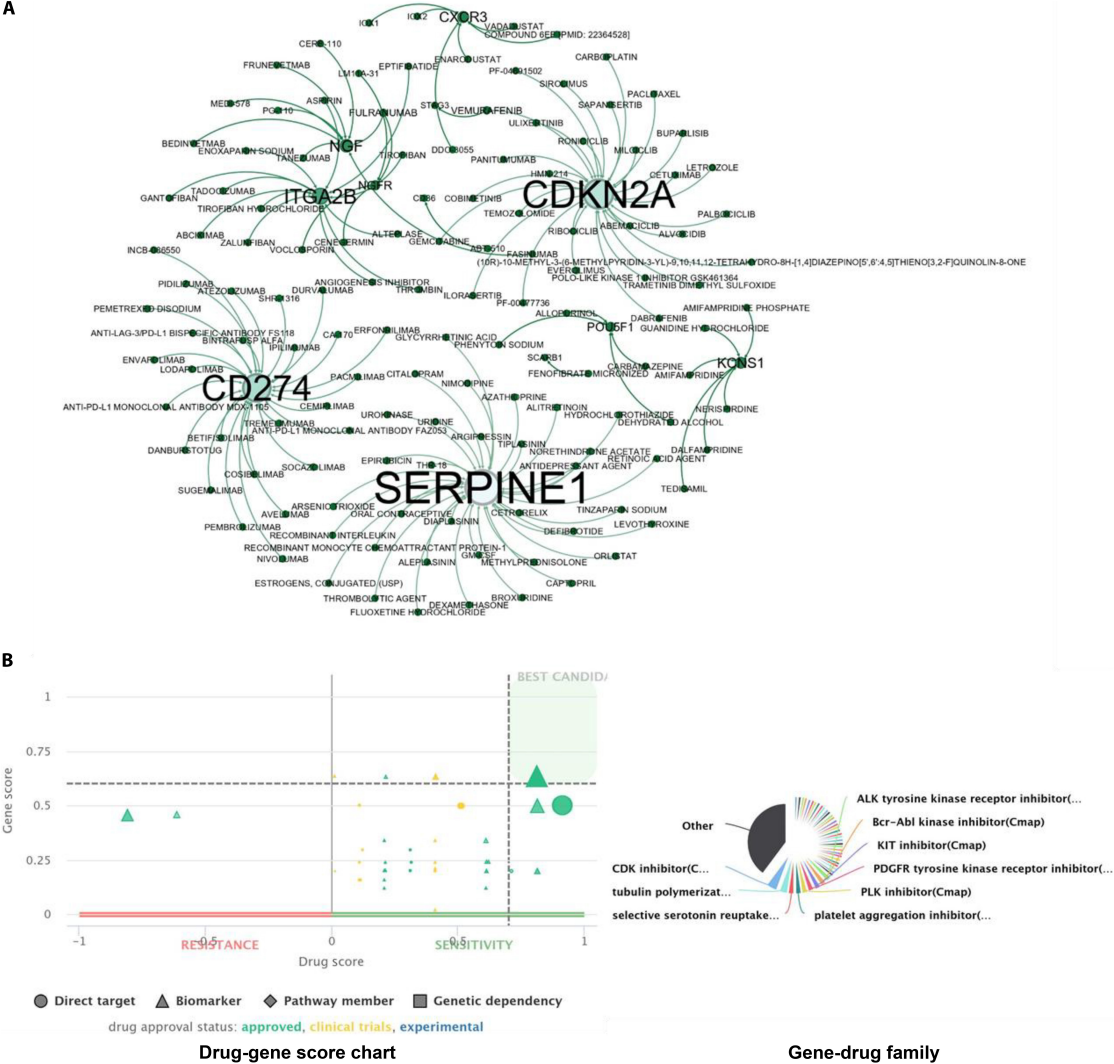
Drug–gene interaction network and prioritization of therapeutic candidates in HPV16-associated cervical cancer. (A) Network visualization of prioritized hub genes and associated drug interactions in HPV16-associated cervical carcinoma. The network integrates disease node-based prioritization of hub genes with drug–gene interactions identified using the Drug-Gene Interaction Database (DGIdb, version 5.0). Disease node scoring was derived from UALCAN expression analysis, and enriched interactions were mapped to highlight druggable targets. Key hub regulators including CDKN2A, SERPINE1, CD274, and CXCR3 show extensive connectivity with multiple FDA-approved or clinically relevant compounds targeting immune regulation, cell cycle progression, and ECM remodeling, emphasizing their potential for targeted or combination therapy in HPV16-driven cancer. (B) Prioritization of therapeutic candidates using PANDrugs analysis. The scatterplot shows GScore (gene relevance) versus DScore (drug suitability) for drug–gene pairs in HPV16-associated cervical cancer. Each point represents a drug interaction categorized as direct target, biomarker, pathway member, or genetic dependency, with color indicating approval status (green: approved; yellow: clinical trials; blue: experimental). The upper-right quadrant highlights high-priority candidates with optimal gene and drug scores. Drug classification analysis indicates a predominance of kinase inhibitors (ALK, Bcr-Abl, KIT, PDGFR, and PLK inhibitors), with additional categories including CDK inhibitors, tubulin polymerization inhibitors, and serotonin reuptake inhibitors, reflecting a diverse spectrum of potential therapeutic options.

To assess the therapeutic relevance of prioritized hub genes, drug–gene interaction profiling was conducted to identify putative therapeutic candidates whose biochemical mechanisms align with the subtype-specific hubs identified in our model. DGIdb was used to identify clinically relevant and FDA-approved antineoplastic agents, as well as additional experimental compounds targeting key disease-associated nodes. This search was complemented by PANDrugs analysis to determine drug family classification and drug/gene scores, supporting the prioritization of compounds for translational potential (Supplementary Data). A total of 20 hub genes were evaluated, revealing multiple actionable interactions, particularly across immune regulation and oncogenic signaling pathways (Fig. 11A and B). These database-reported interactions do not equate to established clinical efficacy in cervical cancer. Instead, they represent mechanistically rational intervention points that serve as a foundation for future drug-repurposing studies. These candidates require independent experimental verification to determine their functional impact within the specific context of the cervical tumor microenvironment.

In HPV16-associated cervical cancer, CD274 (PD-L1) exhibited strong therapeutic relevance, demonstrating direct interactions with immune checkpoint inhibitors such as nivolumab, and anti-CTLA-4 agents including ipilimumab and tremelimumab, all of which are FDA-approved immunotherapies. This supports the central role of immune evasion in HPV16-driven tumorigenesis. Similarly, CXCR3, involved in chemokine-mediated immune cell trafficking, was linked to cytokine and interferon-based modulators, indicating potential immunomodulatory treatment avenues. SERPINE1, a regulator of ECM remodeling and fibrinolysis, demonstrated interaction with small-molecule inhibitors such as tiplaxtinin, suggesting potential application in blocking metastatic spread. Neuroimmune-linked genes NGF and NGFR showed predicted interactions with neuromodulatory compounds, emphasizing their possible roles in tumor–nerve crosstalk. Importantly, CDKN2A, a central tumor suppressor frequently deregulated in HPV-associated carcinogenesis, emerged as a high-value therapeutic node. FDA-approved CDK4/6 inhibitors such as palbociclib, ribociclib, and abemaciclib, which restore p16-mediated tumor suppression by halting CDK-driven cell cycle progression, showed significant interactions with CDKN2A. Additionally, standard chemotherapeutic agents, including carboplatin and paclitaxel, as well as investigational compounds such as roniciclib and milciclib, also interacted strongly with CDKN2A, reinforcing its role as a clinically relevant biomarker and indirect drug target in HPV16-driven cancers. Although transcriptional regulators SOX2, POU5F1 (OCT4), and PRDM14 lacked direct drug interactions, their oncogenic roles underscore the potential for differentiation-based therapies. Notably, docetaxel has been reported to indirectly target SOX2. Collectively, these findings highlight immune checkpoint regulation and ECM remodeling as central therapeutic routes in HPV16-associated malignancies.

**Fig. 12.**
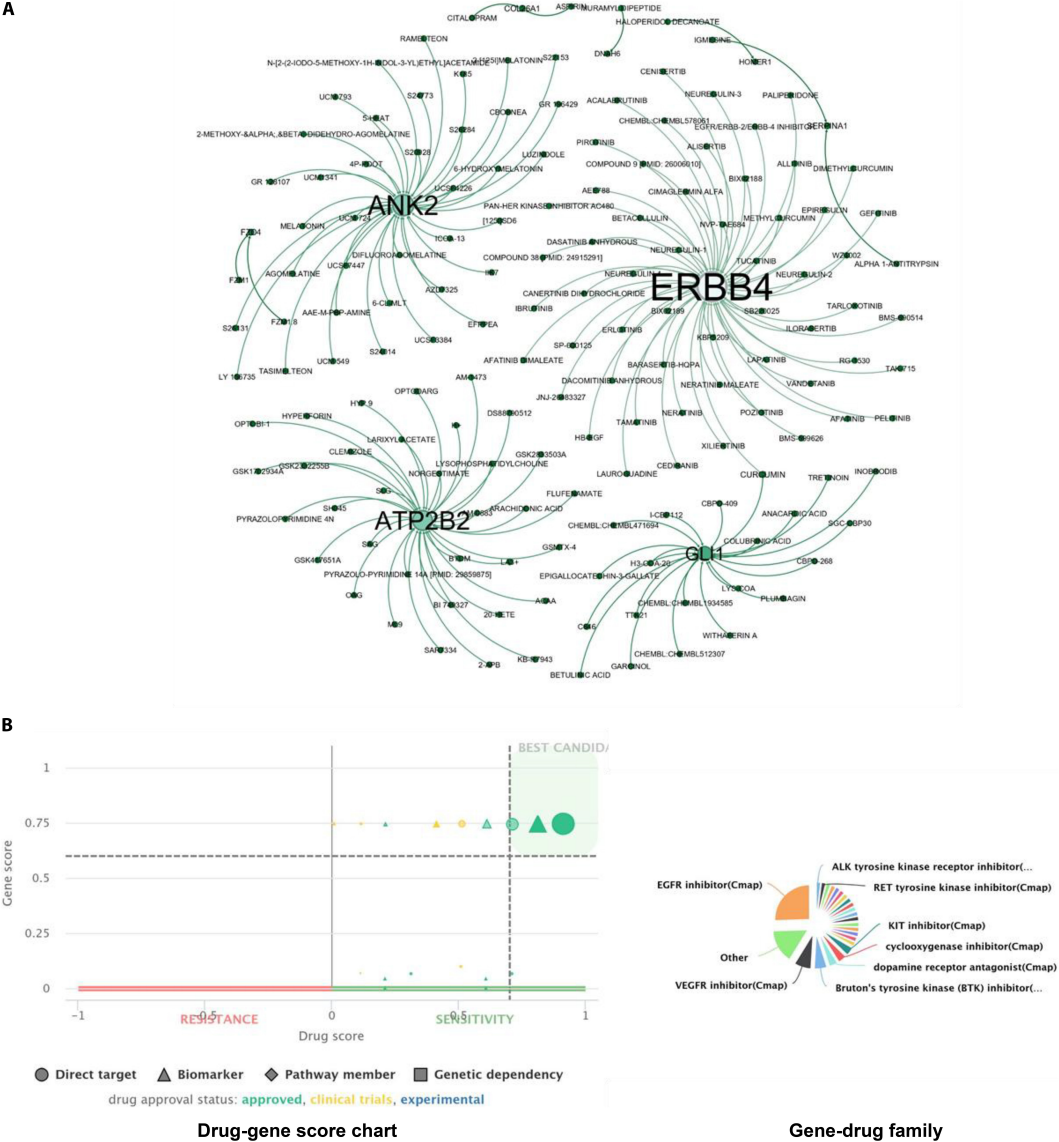
Drug–gene interaction network and prioritization of therapeutic candidates in HPV18-associated cervical cancer. (A) Network visualization of prioritized hub genes and associated drug interactions in HPV18-associated cervical carcinoma. Hub genes were prioritized using disease node scores from UALCAN expression data, and drug–gene interactions were mapped using the Drug-Gene Interaction Database (DGIdb, version 5.0). Key regulators including ERBB4, GLI1, ATP2B2, and ANK2 demonstrate interactions with multiple FDA-approved or clinically relevant compounds targeting oncogenic signaling, calcium transport, and differentiation-associated pathways, suggesting important druggable targets in HPV18-driven carcinogenesis. (B) Prioritization of therapeutic candidates using PANDrugs analysis. The scatterplot displays GScore (gene relevance) versus DScore (drug suitability) for HPV18-associated drug–gene pairs, with each symbol classified as a direct target, biomarker, pathway member, or genetic dependency. Drug approval status is indicated by color (green: approved; yellow: clinical trials; blue: experimental), with high-priority candidates located in the upper-right quadrant. The accompanying pie chart shows pharmacological drug family distribution, highlighting a predominance of epidermal growth factor receptor (EGFR), vascular endothelial growth factor receptor (VEGFR), and other tyrosine kinase receptor inhibitors, underscoring the importance of targeted therapy for HPV18-mediated cervical cancer.

For HPV18-mediated cancer progression, drug–gene interaction analysis of 20 prioritized hub genes (ERBB4, USP37, NLGN2, FZD4, GLI1, HOMER3, ANK2, HOMER1, SHANK1, NOVA1, SALL1, GREB1, COL26A1, NXNL2, DNAJB13, DNAH6, PIGR, SERPINA1, ATP2B2, and NIT1) revealed multiple clinically actionable targets (Fig. 12A and B). Of particular significance, ERBB4, a receptor tyrosine kinase of the EGFR family, demonstrated strong interactions with FDA-approved inhibitors lapatinib, neratinib, and afatinib, all currently used for multiple epithelial malignancies. Additional experimental agents, including cenisertib, pan-HER inhibitor, and cediranib (anti-angiogenic), were also predicted to modulate ERBB4, offering viable repurposing opportunities. GLI1, a transcriptional effector of Hedgehog signaling, showed multiple predicted interactions with synthetic and phytochemical compounds, including garcinol, CBPD-409, TTk21, C646, withaferin-A, plumbagin, curcumin, and epigallocatechin-3-gallate. In light of its reduced expression in TCGA tumor samples, these interactions are interpreted as reflecting Hedgehog pathway involvement at the network and microenvironmental level rather than direct oncogene inhibition, Therefore, we do not imply that GLI1 serves as a direct oncogenic activator in this setting; rather, it highlights pathway-focused modulation as a strictly context-dependent and stage-specific therapeutic concept that requires independent functional validation, highlighting pathway-focused modulation as a context-dependent therapeutic concept requiring functional validation. SERPINA1, involved in inflammation and protease regulation, displayed interactions with small molecules targeting α-1 antitrypsin, suggesting microenvironment-based therapeutic approaches. Conversely, synaptic signaling-associated genes NLGN2, HOMER1, HOMER3, and SHANK1 showed limited pharmacological associations, indicating potential indirect or novel therapeutic angles. Similarly, USP37 and NOVA1 had no known interactions, highlighting new unexplored niches for posttranslational or splicing-targeted therapies. Despite multiple predicted interactions for ANK2 and ATP2B2, their lack of established oncogenic roles in cervical malignancy weakens their current drug prioritization.

Overall, this drug–gene interaction analysis highlights a subset of clinically actionable therapeutic targets, particularly CD274, CDKN2A, and SERPINE1 in HPV16 and ERBB4, Hedgehog pathway-associated signaling, and SERPINA1 in HPV18, offering viable opportunities for drug repurposing, immune checkpoint-based treatments, and targeted pathway inhibition in subtype-specific cervical cancer.

## Discussion

Persistent infection with high-risk HPV types, particularly HPV16 and HPV18, is the leading cause of cervical cancer and is driven by continuous E6 and E7 oncogene activity, which deregulates cell cycle control, suppresses apoptosis, and alters immune and epigenetic responses [35–37]. These viral activities result in keratinization defects, immune evasion, genomic instability, and malignant transformation [7]. Although several studies have identified dysregulated miRNAs in cervical cancer [38,39], the subtype-specific miRNA-mediated regulatory mechanisms of HPV16 and HPV18 remain insufficiently resolved. Consistent with previous studies [19], our results support the role of miRNA dysregulation in HPV-associated cervical carcinogenesis. However, our subtype-specific analysis identified distinct molecular signatures and pathway enrichments associated with individual HPV subtypes. While the degradation of p53 and pRb by E6 and E7 oncoproteins remains the hallmark of cellular transformation, our systems analysis highlights a sophisticated, parallel layer of control: miRNA-mediated host remodeling. Rather than acting as the sole driver of malignancy, the 42 dysregulated miRNAs identified here likely function as fine-tuners of the oncogenic state, warranting further exploration to understand the role in malignancy.

In the present study, initial profiling of HPV-negative and HPV-positive cervical cell lines revealed 42 deregulated miRNAs, among which 4 were further validated using HPV full genome and E6/E7 transfection models. HPV16 infection showed predominant down-regulation of miRNAs, whereas HPV18 infection showed mixed modulation of miRNA expression. These differences may be attributable to variations in epigenetic context and microenvironmental signaling across histotypes [40,41]. Among the deregulated miRNAs, 4 candidate miRNAs (hsa-miR-125b-5p, hsa-miR-106b-5p, hsa-miR-30d-5p, and hsa-miR-23b-3p) were consistently suppressed across all models. Previous studies show that these miRNAs can function as tumor suppressors or regulatory intermediaries within critical pathways, and their suppression may support viral immune escape and uncontrolled proliferation [42–45]. The extent of interference of these miRNA regulations and their regulation by E6 and E7 requires further validation through in vivo HPV models.

Integration of miRNA targets with transcriptomic data demonstrated that loss of miRNA repression contributes to oncogenic gene activation. HPV16-associated target enrichment revealed genes including ZNF541, SYCP2, SMC1B, BRME1, SOX30, and FOXD3-AS1, several of which have emerging relevance in HPV-associated malignancies [46–48]. Shared genes such as TCAM1P, LINC01305, and DMRTA2 represent potential diagnostic markers. Down-regulation of ADAMTS8 in both subtypes supports its suggested tumor-suppressive role [49]. Together, these findings indicate subtype-specific and shared gene regulatory programs.

MF analysis highlighted that HPV16 regulates transcriptional control, oxidoreductase activity, and developmental pathways, likely reflecting keratinocyte lineage plasticity. These computational signatures represent a theoretical potential for HPV16 to modulate cellular oxidative stress, a process essential for oncogenic transformation. In contrast, HPV18 modulated metal-binding, integrin-associated, and GABA receptor-linked signaling. These findings correspond with zinc-dependent E7 mechanisms and alterations in microenvironmental communication [34,50]. Such mechanistic variability may influence treatment response and progression dynamics. This landscape is reinforced by the established role of 17-β-estradiol as a primary co-factor. During HPV infection, a significant association between elevated estradiol levels and the occurrence of cervical squamous cell carcinoma in the context of HPV16 infection has been reported [51]. TThis synergy is further elucidated by a study demonstrating a mutual regulatory loop in which 17-β-estradiol and prolactin (PRL) induce E6/E7 transcript expression, subsequently influencing the expression and nuclear localization of receptors such as ER-α, GPER, and PRLR [52]. Simultaneously, the enrichment of metabolic terms such as androsterone dehydrogenase suggests an emerging role for the androgen/androgen receptor (AR) signaling axis. Androgens play a complex role in the epidemiology and biology of cervical cancer, potentially influencing epithelial differentiation and the tumor microenvironment [53]. The enrichment of metabolic terms like androsterone dehydrogenase and androstan-3-α,17-β-diol dehydrogenase suggests an association with the androgen/AR signaling axis within the HPV16 environment. While high-risk HPV16 typically contributes to cancer progression, recent evidence indicates that the AR may function as a crucial yet understudied “metastatic brake”. Our identification of AR-related metabolic terms and integrin binding aligns with findings that AR activation can suppress cervical cancer cell migration by hindering focal adhesion formation and disrupting the RhoA/ROCK1/LIMK1/CFL1 signaling axis [54].

Pathway analysis further showed that HPV16 deregulates keratinization, metabolic pathways, and transcriptional misregulation, whereas HPV18 primarily affects ECM reorganization and cAMP signaling. cAMP activity has increasingly been recognized as a context-dependent immunosuppressive axis in inflammation and tumor progression [55,56]. Integrating miRNA modulation with these pathway signatures may improve future therapeutic strategies.

Enrichment of transcription-related pathways in our study prompted further examination of TFs associated with HPV-associated cervical cancer, with JARID2 and CBX2 identified as common hub TFs in both HPV16- and HPV18-associated disease. JARID2, an oncogenic regulator, interacts with EZH2 [57,58] and influences epithelial–mesenchymal transition (EMT) via the PTEN/AKT pathway [59], while EZH2 itself can be modulated by miRNAs during HPV infection. Although less studied, CBX2 has been linked to immune evasion through the CBX2–RACK1–HDAC1 complex [60] and has recently been shown to promote cervical cancer progression and cisplatin resistance [61]. Overall, these enriched hub TFs represent key regulatory nodes that warrant further functional investigation, particularly with respect to miRNA-mediated regulation in HPV-associated carcinogenesis.

PPI analysis identified distinct hub genes for each subtype. In HPV16, SOX2, SERPINE1, PRELID2, KCNS1, and KLHL2 exhibited high network scores, indicating strong functional influence. While SERPINE1 and SOX2 are widely studied contributors to oncogenesis [62,63], PRELID2 and KCNS1 highlight potential unexplored mechanisms related to metabolism and ion signaling [64,65]. In HPV18, ERBB4, USP37, FZD4, and GLI1 were top hubs associated with signaling and developmental networks [66,67]. These findings highlight subtype-specific regulatory networks that may inform pathway-oriented precision strategies rather than direct gene-level targeting.

TCGA expression validation supported these trends. HPV16 tumors showed higher expression of SOX2, CD274, CXCR3, PRELID2, and KCNS1, aligning with combined immune evasion and stemness traits [68,69]. HPV18 tumors exhibited significant up-regulation of HOMER3, suggesting altered calcium-dependent signaling [70,71]. These expression differences may contribute to varying clinical outcomes between HPV16 and HPV18 infections.

Drug–gene interaction analyses identified putative repurposable and FDA-approved compounds targeting hub proteins. For HPV16, significant interactions include PD-L1 inhibitors, CDK4/6 inhibitors, and SERPINE1 antagonists. For HPV18, targeting ERBB4 with lapatinib, neratinib, and afatinib, as well as GLI1 inhibition or modulation of the hedgehog pathway with synthetic or phytochemical compounds such as withaferin-A, curcumin, and epigallocatechin gallate (EGCG), represents promising therapeutic avenues [72–75]. These findings support prospects for pathway-focused treatment and drug repurposing. While our network pharmacology analysis identified several natural compounds as putative modulators of HPV-associated hubs, these molecules should be considered experimental leads rather than clinical-grade therapies. Unlike FDA-approved drugs, these phytochemicals often face significant pharmacological challenges, including poor systemic bioavailability, metabolic instability, and nonspecific multi-target effects. Their inclusion in this study demonstrates the mechanistic druggability of the identified regulatory circuits in vitro. Future efforts toward clinical application would require the development of optimized analogs or advanced nanoformulations to overcome these inherent pharmacokinetic limitations.

Overall, this integrative study demonstrates that HPV16 and HPV18 drive cervical cancer through distinct miRNA-mediated molecular networks. HPV16 predominantly reconfigures transcriptional and differentiation programs, whereas HPV18 influences signaling and modulates the tumor microenvironment. Ultimately, our findings suggest that HPV16 and HPV18 are not merely 2 versions of the same threat; they are distinct molecular architects that remodel the host cell using entirely different blueprints. While HPV16 appears to fundamentally “reprogram” the cell’s internal identity and growth cycles, HPV18 acts as a “master communicator”, subverting the signals the cell sends to its neighbors and the surrounding environment. By weaving together our laboratory observations with high-resolution human patient data, we have moved beyond a one-size-fits-all view of cervical cancer. We have identified a unique set of molecular fingerprints for each viral subtype, not as a final clinical solution, but as a newly drafted map for the future.

The regulatory landscape of HPV-associated cervical cancer is increasingly recognized as a multi-layered system. Beyond addressing the requirements for precision and targeted medicine, there is an increasing need for robust, noninvasive screening support systems. While traditional methods rely on a single visual or viral snapshot, systems biology provides a multi-layered resolution. Our findings on the miRNA–target interactome contribute to this evolving systems biology understanding, aligning with recent methodological innovations. For example, the multiple subtypes in one time (MTIOT) framework addresses the historical difficulty of identifying concurrent HPV subtype infections through advanced machine learning integration, providing the high-resolution viral data necessary for precision diagnostics [76]. Similarly, the CITOBOT device standardizes early detection through artificial intelligence (AI)-driven imaging, ensuring robust screening even in resource-limited settings [77]. By mapping the miRNA–target interactome, our study provides a functional link between viral–host interactions and clinical screening tools, uncovering the regulatory disruptions that characterize the transition from oncogenic infection to malignancy. While this regulatory map requires further validation through in vitro functional analysis, in vivo approaches, and clinical trials, it highlights the high-traffic regulatory hubs that must be targeted if we are to move from broad treatment strategies toward truly precise, subtype-specific medicine. The identified subtype-specific biomarkers and drug-associated hub genes therefore establish a foundation for the development of miRNA-assisted precision therapies in cervical cancer.

### Limitations

While our systems-level analysis provides a high-confidence blueprint of the miRNA-mediated HPV-induced host regulatory interface, it is subject to several methodological and biological limitations. First, our framework primarily focuses on the regulatory axis of direct derepression (down-regulated miRNA to up-regulated target). While this serves as a necessary modeling constraint to isolate high-confidence interactions, it represents a simplified snapshot of a dynamic landscape that includes complex feedback loops and indirect transcriptional regulation. Second, the initial discovery phase was restricted to a high-priority 84-miRNA panel; a genome-wide integration would likely reveal a broader range of subtype-specific regulators. In addition, the study primarily incorporates computational prediction and network analysis, with limited experimental validation beyond the expression profiling of 4 selected miRNAs. Functional validation of the predicted miRNA–target interactions and downstream pathway effects would further strengthen the biological conclusions. Third, although clinical validation via GSE151666 and TCGA provided real-world grounding, these datasets were analyzed in aggregate to maximize statistical power for identifying universal regulatory bottlenecks, rather than being stratified by tumor stage or viral integration status. Moreover, TCGA dataset includes a relatively limited number of normal cervical samples compared to tumor samples, which may limit the statistical robustness of the expression comparisons. Finally, the identified regulatory circuits and pharmacological interactions, particularly those involving experimental natural compounds, should be viewed as mechanistic molecular probes rather than established treatments. These candidates require further structural analysis, such as molecular docking, and functional validation to overcome inherent pharmacokinetic hurdles, such as low bioavailability.

## Conclusion

This study provides a comprehensive systems-level perspective on how HPV16 and HPV18 utilize miRNA-mediated regulatory mechanisms to promote cervical carcinogenesis. By integrating data from HPV-positive and HPV-transfected cell models with GEO and TCGA datasets, we demonstrate that HPV16 induces predominant miRNA down-regulation leading to deregulated transcriptional control, altered keratinocyte differentiation, and metabolic perturbations. In contrast, HPV18 influences ECM remodeling, integrin-mediated interaction, and cAMP-linked signaling, emphasizing a distinct dependence on microenvironmental communication. These mechanistic differences were reflected in unique hub gene signatures, including SOX2, SERPINE1, PRELID2, and KCNS1 in HPV16, and ERBB4, FZD4, GLI1, and SERPINA1 in HPV18. Validation of prioritized hubs using TCGA data supports their clinical relevance and underlines subtype-specific traits such as immune evasion, stemness, ligand-driven signaling, and inflammation-associated regulation. Drug–gene interaction analysis further highlighted actionable candidates, including immune checkpoint inhibitors and CDK4/6 inhibitors for HPV16, and ERBB4-targeted therapies alongside Hedgehog pathway-focused modulation strategies for HPV18, suggesting viable avenues for precision therapy and drug repurposing in cervical cancer management. While this study provides strong molecular evidence linking miRNA dysregulation to subtype-specific oncogenic signatures, functional evaluation of newly identified targets, such as PRELID2, KCNS1, USP37, and HOMER3, remains necessary for in-depth mechanistic validation. Similarly, although drug response predictions highlight promising therapeutic leads, preclinical validation in patient-derived models is required to assess efficacy, tumor microenvironment influence, and combinatorial treatment outcomes. Future work addressing these aspects will be essential to translate these molecular insights into clinically applicable strategies for HPV-associated cervical cancer.

## Ethical Approval

No humans or animals were used in this study. All experiments related to this project have been thoroughly reviewed and approved/waived by the Institutional Ethics Committee on Human Research under the Study Protocol No. NARI EC/2024-08.

## Data Availability

The original contributions presented in the study are included in the article/Supplementary Materials. Further inquiries can be directed to the corresponding author.
